# Early prediction of preeclampsia in pregnancy with cell-free RNA

**DOI:** 10.1038/s41586-022-04410-z

**Published:** 2022-02-09

**Authors:** Mira N. Moufarrej, Sevahn K. Vorperian, Ronald J. Wong, Ana A. Campos, Cecele C. Quaintance, Rene V. Sit, Michelle Tan, Angela M. Detweiler, Honey Mekonen, Norma F. Neff, Courtney Baruch-Gravett, James A. Litch, Maurice L. Druzin, Virginia D. Winn, Gary M. Shaw, David K. Stevenson, Stephen R. Quake

**Affiliations:** 1 Department of Bioengineering, Stanford University, Stanford, CA USA; 2 ChEM-H and Department of Chemical Engineering, Stanford University, Stanford, CA USA; 3 Department of Pediatrics, Stanford University School of Medicine, Stanford, CA USA; 4 Chan Zuckerberg Biohub, San Francisco, CA USA; 5 Global Alliance to Prevent Prematurity and Stillbirth (GAPPS), Lynnwood, WA USA; 6 Department of Obstetrics and Gynecology, Stanford University School of Medicine, Stanford, CA USA; 7 Department of Applied Physics, Stanford University, Stanford, CA USA

**Keywords:** Gene expression profiling, Machine learning, Predictive medicine, Predictive markers, Personalized medicine

## Abstract

Liquid biopsies that measure circulating cell-free RNA (cfRNA) offer an opportunity to study the development of pregnancy-related complications in a non-invasive manner and to bridge gaps in clinical care^1–4^. Here we used 404 blood samples from 199 pregnant mothers to identify and validate cfRNA transcriptomic changes that are associated with preeclampsia, a multi-organ syndrome that is the second largest cause of maternal death globally^5^. We find that changes in cfRNA gene expression between normotensive and preeclamptic mothers are marked and stable early in gestation, well before the onset of symptoms. These changes are enriched for genes specific to neuromuscular, endothelial and immune cell types and tissues that reflect key aspects of preeclampsia physiology^6–9^, suggest new hypotheses for disease progression and correlate with maternal organ health. This enabled the identification and independent validation of a panel of 18 genes that when measured between 5 and 16 weeks of gestation can form the basis of a liquid biopsy test that would identify mothers at risk of preeclampsia long before clinical symptoms manifest themselves. Tests based on these observations could help predict and manage who is at risk for preeclampsia—an important objective for obstetric care^10,11^.

## Abstract (web summary)

Analyses of circulating cell-free RNA (cfRNA) in blood samples from pregnant mothers identify changes in gene expression that could be used in liquid biopsy tests to identify and monitor individuals who are at risk of preeclampsia.

## Main

Advances in obstetrics and neonatology have substantially mitigated many of the adverse pregnancy outcomes related to preterm birth and preeclampsia^3^. Nonetheless, the standard of care implemented today focuses on how to treat a mother and child once a complication has been diagnosed, which proves both insufficient and costly^1,2,4,12^: Preeclampsia and related hypertensive disorders cause 14% of maternal deaths each year globally, second only to haemorrhage^5^, and cost nearly US$2 billion in care in the first year following delivery^2^. Worse, three out of five maternal deaths in the USA are preventable and often associated with a missed or delayed diagnosis^13^. Such outcomes highlight the need for tools that would aid in identifying which mothers are at risk for preeclampsia before clinical presentation^10,11^.

Formally defined as new-onset hypertension with proteinuria or other organ damage (for example, renal, liver or brain) occurring after 20 weeks of gestation^14^, preeclampsia can clinically manifest anytime thereafter, including into the post-partum period^15^. So far, no recommended test exists that can predict the future onset of preeclampsia early in pregnancy^10^. Liquid biopsies that measure plasma cfRNA suggest a means to achieve this^16^; there have been promising results both in the confirmation of preeclampsia at clinical diagnosis^17,18^ and earlier in pregnancy^19^. The prediction of preeclampsia early in gestation, before symptoms present, could guide the prophylactic use of potential therapeutic agents^10^ such as low-dose aspirin^11^.

Preeclampsia is specific to humans^6^ and a few non-human primates^7^, and consequently, elucidating its pathogenesis has proven challenging. Broadly, it is accepted that preeclampsia occurs in two stages—abnormal placentation early in pregnancy followed by systemic endothelial dysfunction^6,8,9^. Preeclampsia can present with a diversity of symptoms and efforts to subclassify the disease on the basis of the timing of onset^20^ have had mixed success^6,9,21,22^. Separate efforts have focused on subtyping preeclampsia molecularly using placental gene expression and histology^23,24^. As cfRNA is derived from many tissues in the body^25,26^, liquid biopsies present a potential means to indirectly observe pathogenesis in real time and to identify physiological changes associated with preeclampsia for proposed subtypes.

Here we report that cfRNA transcriptomic changes can distinguish between normotensive and preeclampsia pregnancies throughout the course of pregnancy, irrespective of preeclampsia subtype. The majority of these cfRNA changes are most marked early in pregnancy—well before the onset of symptoms. Neuromuscular, endothelial and immune cell types and tissues contribute to these cfRNA changes, consistent with important aspects of the pathogenesis of preeclampsia and also suggesting new approaches to stratify the disease. These observations enabled us to identify and independently validate a panel of 18 genes that when measured between 5 and 16 weeks of gestation form a predictive signature of preeclampsia risk. cfRNA measurements also reflect the multifactorial nature of preeclampsia and provide a means to monitor maternal organ health in a non-invasive manner. Together, these results show that cfRNA measurements can form the basis for clinically relevant tests that would predict preeclampsia months before presentation, manage who is at risk for specific organ damage and help to characterize the pathogenesis of preeclampsia in real time.

### Clinical study design

**Fig. 1:**
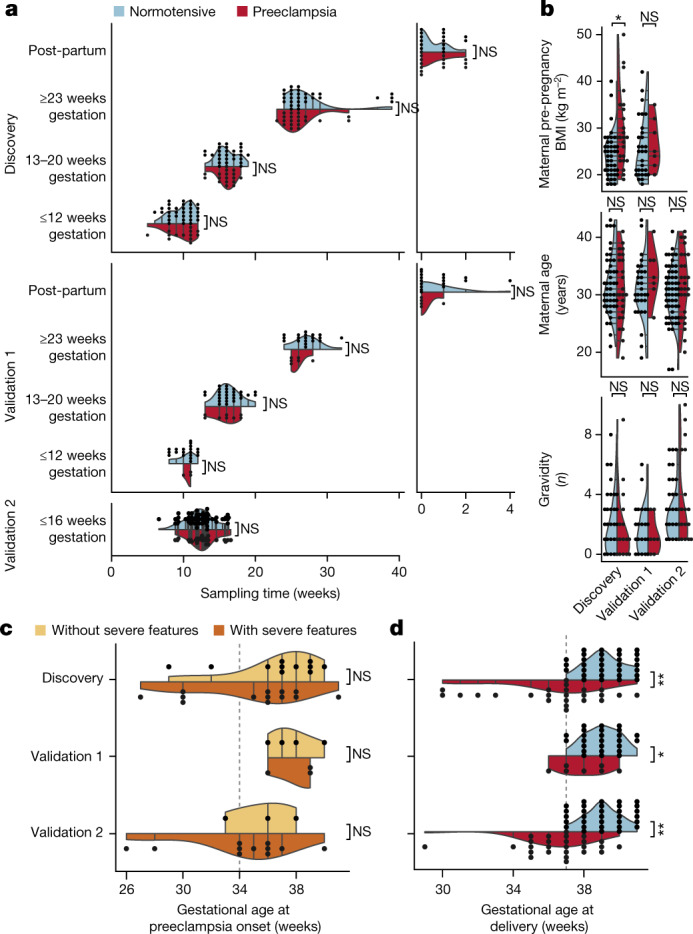
Comparing sample, maternal and pregnancy characteristics for normotensive and preeclampsia groups across cohorts. **a**, Matched sample collection time both across gestation (left) and after delivery (right). **b**, Maternal characteristics (*P* = 0.02 comparing BMI in the discovery cohort). **c**, Matched gestational age at preeclampsia onset regardless of preeclampsia symptom severity. **d**, Gestational age at delivery (*P* = 6 × 10^−7^, 0.04, 8 × 10^−9^) for the discovery (*n* = 49 normotensive [37, 36, 39, 30]; *n* = 24 with preeclampsia [13, 17, 20, 17]), validation 1 (*n* = 32 normotensive [19, 27, 19, 19]; *n* = 7 with preeclampsia [3, 8, 6, 5]) and validation 2 (*n* = 61 normotensive [61]; 26 with preeclampsia [28]) cohorts. Square brackets indicate the sample number per collection group. For **a**, statistics were calculated by sample group. For **b**–**d**, statistics were calculated by cohort group (NS = not significant, *P < 0.05, **P ≤ 10^−7^; two-sided (**a**–**c**) and one-sided (**d**) Mann–Whitney rank test).

To identify changes associated with preeclampsia well before traditional diagnosis, we designed a prospective study and recruited pregnant mothers at their first clinical visit to Stanford’s Lucile Packard Children’s Hospital. For each participant, we analysed cfRNA for samples collected before or at 12 weeks, between 13 and 20 weeks, at or after 23 weeks of gestation and post-partum. We then split this larger group into discovery (*n* = 73, (49 normotensive, 24 with preeclampsia)) and validation 1 (*n* = 39, (32 normotensive, 7 with preeclampsia)) cohorts. We also obtained samples from an independent cohort of 87 mothers (validation 2); these samples were collected at several separate institutions before 16 weeks of gestation (61 normotensive, 26 with preeclampsia) (Fig. 1a).

**Extended Data Fig. 1:**
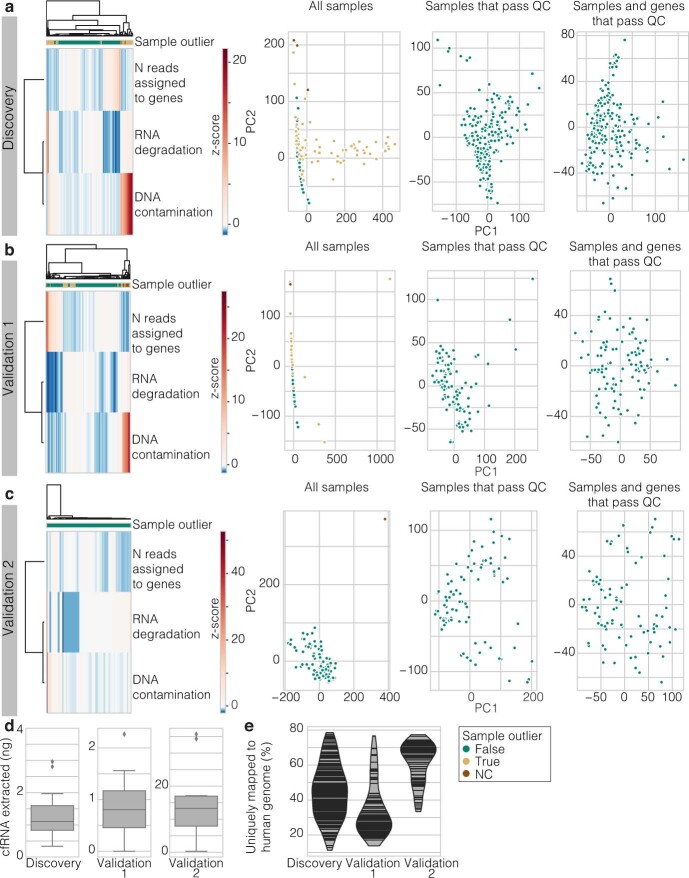
Samples with outlier values for at least one QC metric cluster separately from most non-outlier samples. **a**–**c**, For discovery (**a**), validation 1 (**b**), and validation 2 (**c**), hierarchical clustering (left) and PCA (right) reveals that most outlier samples cluster with negative control (NC) samples and separately from non-outlier samples. **d**, **e**, Visualization of other QC metrics like the amount of cfRNA extracted (**d**) and the percent of reads that align uniquely to the human genome (**e**) (n = 209, 106, 89 samples for discovery, validation 1, and validation 2, respectively). For PCA in **a**–**c**, sample outliers and poorly detected genes drive PCA and serve as leverage points. The top two principal components are visualized when performed using all samples and all genes (leftmost PCA) or only samples that pass QC metrics (middle PCA) reveals that certain samples can act as leverage points. Once sample outliers and lowly detected genes are removed from the cfRNA gene matrix (rightmost PCA), the top two principal components reflect natural variance in the data and are no longer driven by a few leverage points. For box plots, centre line, box limits, whiskers and outliers represent the median, upper and lower quartiles, 1.5× interquartile range and any outliers outside that distribution, respectively.

All cohorts included individuals of diverse racial and ethnic backgrounds in approximately matched proportions across normotensive and preeclampsia groups (Extended Data Table 1). A pregnancy was considered to be normotensive if it was both uncomplicated and went to full-term (37 or more weeks), or as preeclampsia with or without severe features on the basis of current guidelines (see Methods). For mothers who developed preeclampsia, all antenatal blood samples were collected before diagnosis. Our final analysis included a subset of those samples that passed predefined quality metrics (Extended Data Fig. 1, Supplementary Note 1, Supplementary Table 1, Methods).

Across gestational time points in all cohorts, we found no significant difference in sampling time between preeclampsia and normotensive groups (*P* ≥ 0.26, 0.11, 0.46) (values are reported as discovery, validation 1, validation 2; two-sided Mann–Whitney rank test unless otherwise specified). Known risk factors for preeclampsia, such as pre-pregnancy maternal body mass index (BMI), maternal age and gravidity followed expected trends. BMI was significantly different between preeclampsia and normotensive groups in the discovery cohort alone (*P* = 0.02, 0.45, not available), whereas maternal age and gravidity were not (*P* ≥ 0.29, 0.16, 0.2) (Fig. 1b, Extended Data Table 1). In validation 2, history of preterm birth and mode of delivery were significantly different between normotensive and preeclampsia groups. Other demographic factors such as race, ethnicity and nulliparity differed across cohorts but not between case groups within each cohort (*P* ≤ 0.05, two-sided chi-squared test for categorical or ANOVA for continuous variables with Bonferroni correction; Extended Data Table 1, Supplementary Table 2).

In mothers who later developed preeclampsia, we observed no significant difference (*P* = 0.14, 1.0, 0.4) in gestational age at onset between those who did not experience severe symptoms (*n* = 11, 4, 3*) and those who did experience severe symptoms (*n* = 13, 3, 13*) (*denotes incomplete data for the specified cohort) (Fig. 1c). Furthermore, 21 mothers who developed preeclampsia also delivered preterm (*n* = 9, 1, 11) as compared with no mothers in the normotensive group; this was reflected by significantly different gestational ages at delivery (*P* = 10^−7^, 0.04, 10^−9^; one-sided Mann-Whitney rank test) (Fig. 1d) and lower fetal weight at delivery (Extended Data Table 1), which is consistent with epidemiological evidence that preeclampsia increases the risk of spontaneous or indicated preterm delivery^2,27^.

### Identifying preeclampsia-related cfRNA changes

**Fig. 2:**
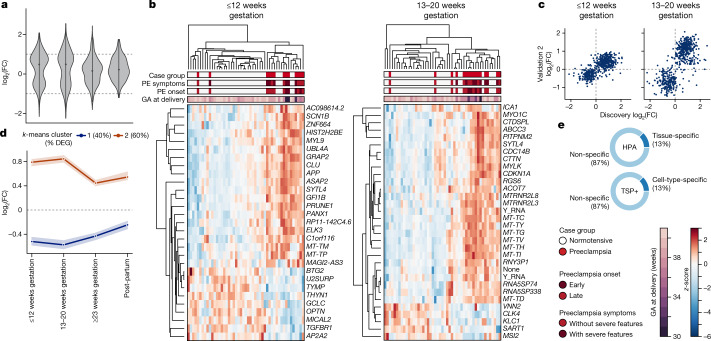
Before 20 weeks of gestation, cfRNA measurements segregate preeclampsia and normotensive samples and are enriched for neuromuscular, endothelial and immune cell types and tissues. **a**, Distribution of log﻿_2_(FC) for DEGs (*n* = 544) with dashed lines at log﻿_2_(FC) = ±1. **b**, Before 20 weeks of gestation, a subset of DEGs can separate ﻿preeclampsia (PE) and normotensive samples despite differences in symptom severity, preeclampsia onset subtype and gestational age (GA) at delivery. *HIST2H2BE* is also known as *H2BC21*. See Supplementary Table 3 for more information on genes included in heatmaps. **c**, Comparison of log﻿_2_(FC) for DEGs between the discovery and the validation 2 cohorts reveals strong agreement. **d**, DEGs for preeclampsia as compared to normotensive samples can be described as either increased (orange) or decreased (dark blue) in preeclampsia over gestation. Points indicate median per trend and shaded region indicates 95% CI. **e**, Approximately 13% of DEGs are tissue- or cell-type-specific when compared with the Human Protein Atlas (HPA) and an augmented Tabula Sapiens (TSP+) atlas.

**Extended Data Fig. 2:**
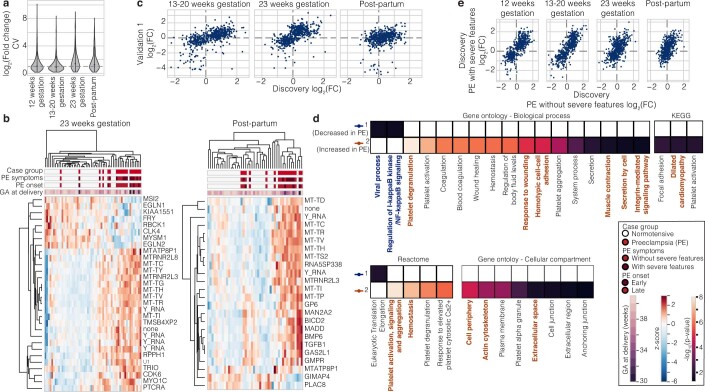
Across gestation before diagnosis, changes in the cfRNA transcriptome segregate preeclampsia and normotensive samples and reflect known preeclampsia biology. **a**, Distribution of CVs with dashed line at CV = 1 for all DEGs (n = 544) between preeclampsia as compared to normotensive samples across gestation. **b**, At ≥23 weeks of gestation and post-partum, a subset of DEGs can separate preeclampsia (n = 20, 17) and normotensive (n = 37, 29) samples despite differences in symptom severity, preeclampsia onset subtype, and GA at delivery. **c**, Comparison of log﻿_2_(FC) for DEGs for preeclampsia as compared to normotensive between discovery and validation 1 reveals good agreement across gestation but not post-partum. **d**, The genes in each longitudinal trend group reflect known preeclampsia aetiology as highlighted across four databases (GO biological processes, KEGG, the reactome, and GO cellular compartment). Some preeclampsia associated terms are emphasized in bold, coloured text that corresponds to group colour from Fig. 2d (Dark blue and orange indicate decreased and increased in preeclampsia versus normotensive, respectively) (p ≤ 0.05; one-sided hypergeometric test with multiple hypothesis correction, see Methods). **e**, Comparison of ﻿log_2_(FC) for DEGs for preeclampsia without severe features versus normotensive and preeclampsia with severe features versus normotensive in the discovery cohort reveals good agreement along the y=x axis with a slope of 0.93, 1.03, 0.77, and 0.86 at ≤12 weeks, 13–20, ≥23 weeks, and post-partum, respectively.

A total of 544 differentially expressed genes (DEGs) were altered across gestation and post-partum between mothers who later developed preeclampsia with or without severe features and normotensive mothers who did not experience complications (*P* ≤ 0.05; see Methods). Most DEGs were annotated as protein-coding and a small fraction (43; 8%) were other types, including 11 mitochondrial transfer RNAs, 6 long non-coding RNAs, 8 pseudogenes and 1 small nucleolar RNA (snoRNA). These changes in gene expression occurred most notably before 20 weeks of gestation, as indicated by a clear bimodal distribution with two peaks centred around a log_2_-transformed fold change (log﻿_2_(FC)) of +0.8 and −0.6 (Fig. 2a). Changes in gene expression were also most stable before 20 weeks of gestation, at which point over 50% of genes had a coefficient of variation (CV) < 1 as compared to 31% of genes at or after 23 weeks of gestation and 36% at post-partum (Extended Data Fig. 2a).

**Extended Data Fig. 3:**
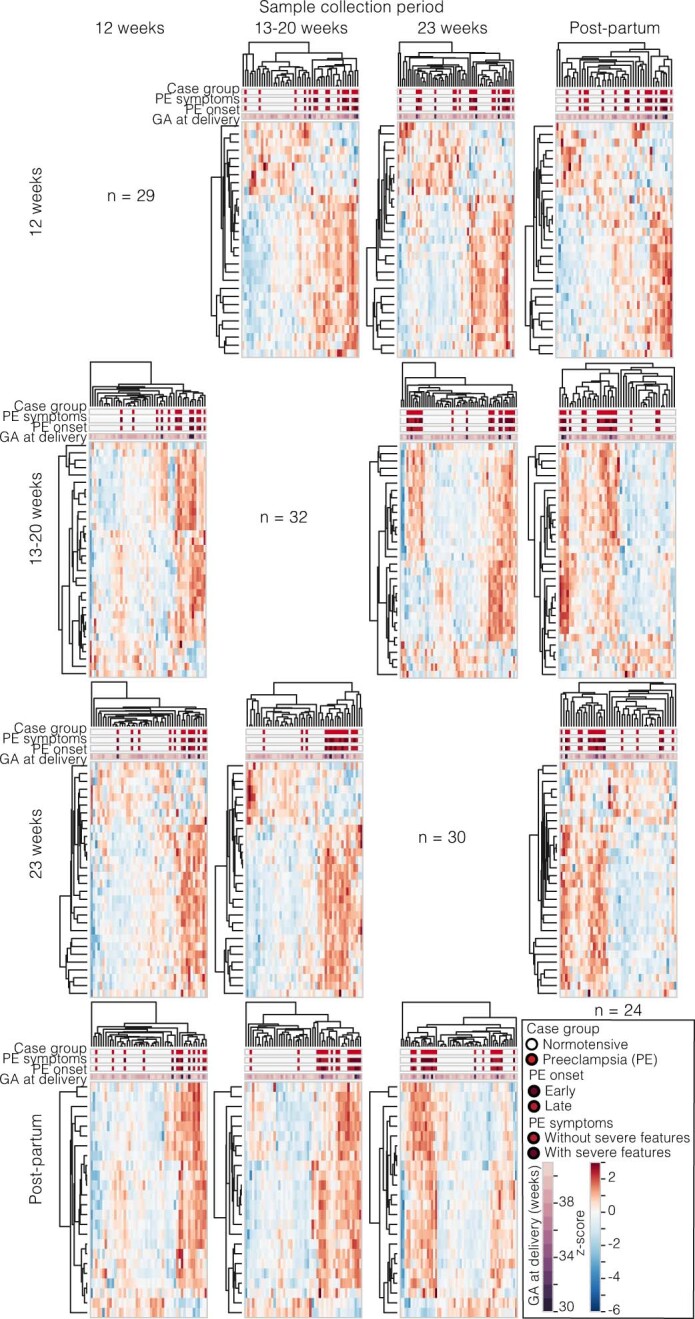
Across gestation and before diagnosis, changes in the cfRNA transcriptome identified at one time point can moderately segregate preeclampsia and normotensive samples at other time points. DEGs with |﻿log_2_(FC)| ≥ 1 and CV < 0.5 or 0.4 for the 13–20 week time point were identified at each time point across gestation. Each row visualizes how well a specific DEG subset from a given sample collection period can separate preeclampsia (n = 13, 16, 20, 17) and normotensive (n = 36, 33, 37, 29) samples in all other collection periods (≤12, 13–20, ≥23 weeks of gestation and post-partum respectively). The number of genes identified per sample collection period is highlighted along the main diagonal.

We then asked whether a subset of genes approximately proportional in number to the total sample number (*n* = 49, 49, 57, 46 for 12 weeks or less, 13–20 weeks, 23 weeks or more of gestation, and post-partum, respectively) was sufficient to segregate preeclampsia (*n* = 13, 16, 20, 17) and normotensive (*n* = 36, 33, 37, 29) samples across gestation. We found that 24–32 genes were sufficient to separate preeclampsia and normotensive samples across gestation and at post-partum with good specificity (86% [75–93%], 79% [66–88%], 97% [90–100%] and 90% [78–96%]) and sensitivity (85% [64–95%], 88% [69–96%], 65% [47–80%] and 71% [51–86%]) (values in square brackets are 90% confidence intervals (CI); Fig. 2b, Extended Data Fig. 2b). See also Extended Data Fig. 3, Supplementary Table 3.

Nearly all 544 DEG changes showed strong agreement in both validation cohorts as compared to the discovery cohort across gestation but not post-partum. Specifically, more than 82% and 92% of genes across gestation had the same log﻿_2_(FC) sign, with a Spearman correlation of at least 0.67 and 0.71 for validation 1 and validation 2 respectively (*P* < 10^−15^; two-sided *t*-test) as compared with 60% and 0.35 post-partum (Fig. 2c, Extended Data Fig. 2c). Finally, we asked whether symptom severity correlated with ﻿log_2_(FC) magnitude for these 544 DEGs common to both preeclampsia subtypes. We found that on average, symptom severity did not influence ﻿log_2_(FC) magnitude as reflected by a slope of nearly one across gestation (Extended Data Fig. 2e).

### cfRNA changes reflect preeclampsia pathophysiology

**Extended Data Fig. 4:**
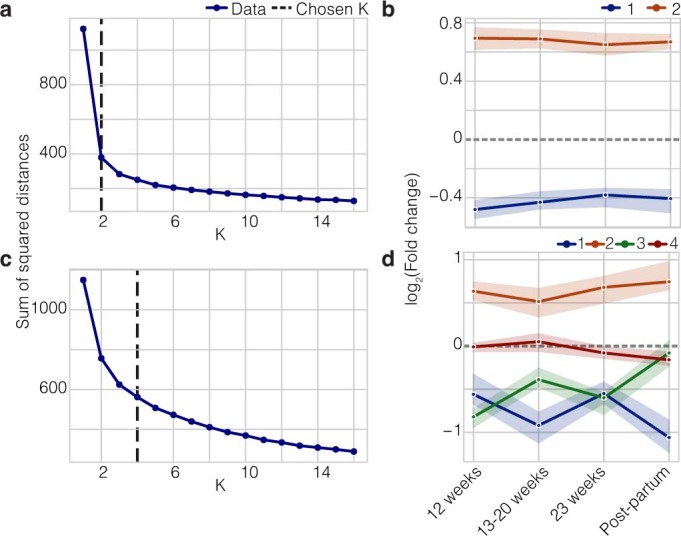
*k*-means clustering reveals meaningful longitudinal patterns. **a**, **c**, The chosen *k* clusters (dashed line) comparing a performance metric, the sum of squared distances, and values of *k* for clustering of DEGs for preeclampsia versus normotensive related to Fig. 2d (**a**) and DEGs for preeclampsia with versus without severe features related to Fig. 4a (**c**). **b**, **d**, Following permutation of the data matrix prior to *k*-means clustering, longitudinal changes over gestation are replaced by 2 flat lines for clustering of ﻿log_2_(FC) for preeclampsia versus normotensive (**b**) and 4 uninformative lines for clustering of ﻿log_2_(FC) for preeclampsia with versus without severe features (**d**). For **b**, **d**, points indicate median per trend and shaded region indicates 95% CI.

The 544 identified DEGs could be well categorized into two longitudinal trends (Fig. 2d, Extended Data Fig. 4a, c). Resembling a V shape, the first trend (group 1) described the longitudinal behaviour of 216 genes (40%), for which measured levels were reduced in preeclampsia samples (−1.3× to −1.5×) across gestation with a minimum between 13 and 20 weeks. Peaking in early gestation before 20 weeks (1.75×), the second trend (group 2) described the behaviour of 328 genes (60%) that had significantly increased levels in preeclampsia samples before 20 weeks and to a lesser extent after 23 weeks of gestation (1.3×). For group 1 but not group 2, gene changes were far less evident post-partum and trended towards no difference between preeclampsia and normotensive, which may reflect a placental contribution. DEGs were also enriched for genes previously implicated in preeclampsia^28^ broadly (30 gene overlap, *P* = 0.006; one-sided hypergeometric test) and more specifically through placental biopsies^23,24^ (two of nine previously identified genes overlap, *PIK3CB*, *TAP1*) (Extended Data Table 2).

Approximately 13% of DEGs were tissue- or cell-type-specific (Fig. 2e). Genes that were decreased in preeclampsia across gestation (group 1) were broadly enriched for the immune system, whereas those genes increased in preeclampsia across gestation (group 2) were enriched for nervous, muscular, endothelial and immune contributions as reflected by cell type and pathway enrichment (*P* ≤ 0.05; one-sided hypergeometric test with multiple hypothesis correction, see Methods) (Extended Data Fig. 2e, Extended Data Table 2, Supplementary Table 4). Consistent with the known pathogenesis of preeclampsia, we identified a strong endothelial-linked signal underscored by contributions from capillary aerocytes (*P* = 0.03), an endothelial cell type specific to the lungs^29^, platelets (*P* = 10^−33^) and several platelet-related pathways like platelet degranulation (*P* = 10^−9^) and platelet activation, signalling and aggregation (*P* = 10^−8^) among others. We also identified a small, borderline-significant placental contribution (*P* = 0.16) from two genes (*IGF2*, *TGM2*) in group 1 with established roles in trophoblast development^30,31^.

We found increased nervous and muscular contributions for preeclampsia as emphasized by contributions from excitatory neurons (*P* = 0.02), oligodendrocytes (*P* = 0.005) and smooth muscle (*P* = 0.0003), and terms like muscle contraction﻿ (*P* = 0.02) and dilated cardiomyopathy (*P* = 0.01). The immune system also contributes to both increased (for example, mesenchymal stem cells, total peripheral blood mononuclear cells) and decreased (granulocytes, T cells) changes across gestation. Genes in both groups were enriched for signalling pathways (that is, secretion by cell, integrin-mediated signalling pathway, regulation of IκB kinase, NF-κB signalling). Group 2 was also enriched for cellular compartments such as the cell periphery, cell junctions and extracellular space, consistent with reports that preeclampsia may be associated with signalling from the fetoplacental complex^32^.

### Risk prediction early in gestation

**Fig. 3:**
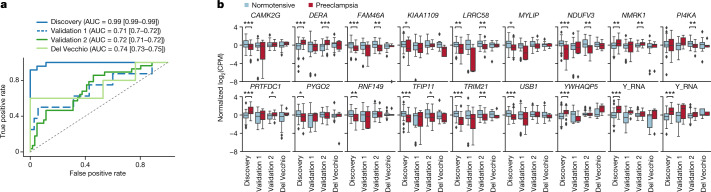
A subset of cfRNA changes can predict risk of preeclampsia early in gestation. **a**, Classifier performance as quantified by receiver operator characteristic curve (ROC) for samples collected in early gestation between 5 and 16 weeks, with AUROC and corresponding 90% CI noted per cohort. **b**, Prediction of preeclampsia incorporates cfRNA levels for 18 genes for which normalized centred log_2_(FC) trends hold across the discovery (*n* = 61 normotensive, 24 preeclampsia), validation 1 (*n* = 35 normotensive, 8 preeclampsia), validation 2 (*n* = 61 normotensive, 28 preeclampsia) and Del Vecchio (*n* = 17 normotensive or other complication, 5 preeclampsia) cohorts as confirmed using univariate analysis (*P ≤ 0.05, **P ≤ 0.01, ***P ≤ 0.005; one-sided Mann–Whitney rank test with Benjamini–Hochberg correction). See Supplementary Table 5 for exact *P* values. For box plots, centre line, box limits, whiskers and outliers represent the median, upper and lower quartiles, 1.5× interquartile range and any outliers outside that distribution, respectively. Plot limits are −8 to 4 to better visualize the main distribution. log_2_(CPM), log_2_-transformed counts per million reads.

As gene expression changes associated with preeclampsia pathogenesis across gestation were readily detected irrespective of symptom severity, we sought to build a classifier that could identify mothers at risk of preeclampsia at or before 16 weeks of gestation (Extended Data Fig. 2e, Supplementary Note 2). We trained a logistic regression model on the discovery cohort (*n* = 61 normotensive, 24 with preeclampsia). After training, the final model performed well, with a near-perfect area under the receiver operating characteristic curve (AUROC) (0.99 [0.99–0.99]), good specificity (85% [77–91%]) and perfect sensitivity (100% [92–100%]) (Fig. 3a, Extended Data Table 3). We then tested this model on validation 1 (*n* = 35 normotensive, 8 preeclampsia) and two other independent cohorts, which were collected at separate institutions: validation 2 (*n* = 61 normotensive, 28 preeclampsia) and the cohort used by Del Vecchio and colleagues^19^ (*n* = 8 normotensive, 5 with preeclampsia, 7 with gestational diabetes, 2 with chronic hypertension). Across these cohorts, the final model once again performed well, with consistent AUROC﻿ (0.71 [0.70–0.72], 0.72 [0.71–0.72], 0.74 [0.73–0.75]), sensitivity (75% [46–92%], 56% [42–72%], 60% [26–87%]) and specificity (56% [43–70%], 69% [59–78%], 100% [89–100%]) (all reported as validation 1, validation 2, Del Vecchio, value [90% CI]) (Fig. 3a, Extended Data Table 3).

**Extended Data Fig. 5:**
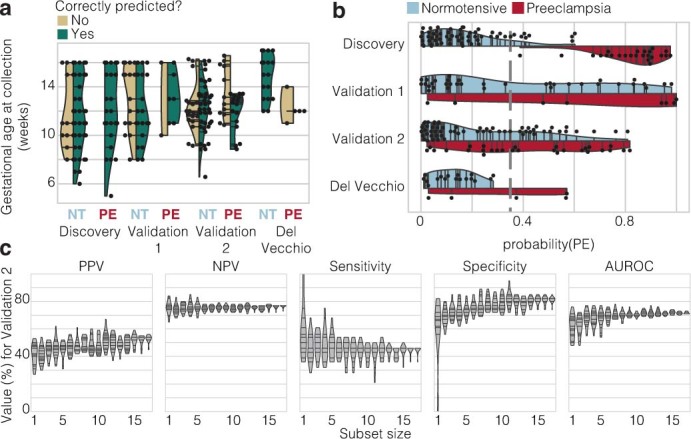
Examining the logistic regression model used to predict risk of preeclampsia early in gestation. **a**, Comparison of gestational age at sample collection (weeks) for incorrectly predicted (yellow) or correctly predicted (green) samples across normotensive and preeclampsia groups in the discovery, validation 1, validation 2 and Del Vecchio cohorts shows that incorrectly predicted preeclampsia samples (false negatives) are collected at later gestational ages. **b**, Estimated probability of preeclampsia as outputted by logistic regression for both preeclampsia and normotensive samples shows that the model is well-calibrated across most predictions. Dashed line at 0.35 indicates classifier cut-off where *P*(PE) ≥ 0.35 constitutes a sample predicted as preeclampsia. **c**, Logistic regression models trained on subsets of 1–18 genes of the initial 18 genes can moderately predict future preeclampsia onset in the validation 2 cohort with improving performance as subset size increases and as characterized by PPV, NPV, sensitivity, specificity and AUROC (left to right).

Misclassified individuals did not predominantly belong to one racial or ethnic group; rather, the fraction of misclassified individuals for each race and ethnicity matched the cohort distribution as a whole. Notably, this holds true even for a group not included in the discovery cohort (American Indian or Alaskan Native) which made up 23% of both case and control for the validation 2 cohort. For false negatives in validation 2 and Del Vecchio, we find a shift to later gestational ages at collection (13.5 ± 2, 12.5 ± 2 weeks) as compared to preeclampsia samples that were correctly classified (12 ± 2, 12 ± 0 weeks; mean ± s.d. for validation 2, Del Vecchio) (Extended Data Fig. 5a). This suggests that in practice, there may an optimal collection window to reduce false negatives. Indeed, if we only consider samples before 14 weeks of gestation, we observe a 9% and a 15% increase in sensitivity, with corresponding AUROC values of 0.73 and 0.90 for validation 2 and Del Vecchio, respectively. There were no false positives from the Del Vecchio cohort, suggesting that the model can distinguish between preeclampsia and other risks like chronic hypertension or gestational diabetes. The model also proved well-calibrated, estimating a slightly increased probability of preeclampsia for gestational diabetes (0.15 ± 0.08) and chronic hypertension (0.18 ± 0.13)—known preeclampsia risk factors^33,34^—as compared to the estimate for normotensive samples (0.1 ± 0.08) (mean ± s.d., Extended Data Fig. 5b). These increased probabilities for other risk factors affected the AUROC﻿ of the test (0.74 [0.73–0.75] as compared to 0.8 [0.79–0.81] for only preeclampsia versus normotensive samples, Extended Data Table 3) (all reported as value [90% CI]).

Finally, we inspected the 18 genes (Fig. 3b, Extended Data Table 4) used by the model to yield probability estimates. Eight genes were annotated in the Human Protein Atlas (HPA, v.19)^35^ as enhanced or enriched in the placenta (*TENT5A* (also known as *FAM46A*) and *MYLIP*), neuromuscular (*CAMK2G*, *NDUFV3*, *PI4KA* and *PRTFDC1*) and immune system (*RNF149* and *TRIM21*). Univariate analysis further confirmed that nine of the gene trends (that is, decreased or increased gene levels in preeclampsia) observed in the discovery dataset are upheld in validation 2 (*P* ≤ 0.05; one-sided Mann–Whitney rank test with Benjamini–Hochberg correction) (Fig. 3b, Supplementary Table 5). We also found that most models trained using a subset of the 18 initial genes can predict future preeclampsia onset with varying performance. Notably, performance improved across all metrics (sensitivity, specificity and AUROC) as we increased the number of genes included for model training (Supplementary Table 6, Extended Data Fig. 5c).

### Preeclampsia as a multifactorial disease

**Fig. 4:**
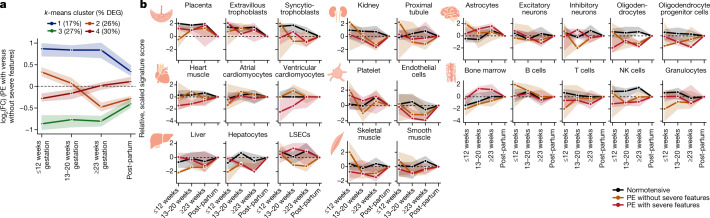
cfRNA measurements reflect the multifactorial nature and pathogenesis of preeclampsia over pregnancy before diagnosis. **a**, DEGs for preeclampsia with as compared to without severe features (*n* = 503) can be described by four longitudinal trends. **b**, Comparison of organ and cell-type changes over gestation for eight organ systems reflects the multifactorial nature of preeclampsia and provides a possible means to comprehensively monitor maternal organ health. Points indicate median per sample group and shaded region indicates 95% CI in **a** and 75% CI in **b**. LSECs, liver sinusoidal endothelial cells; NK cells, natural killer cells.

By comparing mothers who later developed preeclampsia with or without severe symptoms, we identified 503 DEGs (*P* ≤ 0.05). As there were no significant differences in symptom severity as related to the timing of preeclampsia onset (Fig. 1c), we believe that our observations contrasting preeclampsia with and without severe symptoms are not obscured by differences in preeclampsia-onset type. DEGs could be well categorized into four longitudinal trends (Fig. 4a, Extended Data Fig. 4b, d). Two groups (groups 1 and 3) described the temporal behaviour of 217 genes (44%), for which measured levels were either consistently increased (group 1) or reduced (group 3) in preeclampsia with as compared to without severe symptoms (±1.8×) across gestation and trended towards no change post-partum. By contrast, groups 2 and 4 (286 genes, 56%) changed signs in mid-gestation, beginning as slightly increased (group 2, 1.2×) or decreased (group 4, −1.2×) in severe preeclampsia and then moving to decreased (group 2, −1.4×) or unchanged (Group 4, 1×) at 23 weeks or more of gestation.

Analysis of the enriched cell types and tissues of origin for each of these groups revealed that increased gene differences in severe preeclampsia were driven by contributions from endothelial cells and the adaptive immune system (bone marrow). By contrast, genes that changed signs over gestation were enriched for innate immune cell types (for example, granulocytes and neutrophils for group 2, thymus for group 4) (Extended Data Table 5). Quantifying the total cfRNA signal confirmed an increased bone marrow signal for only severe preeclampsia across gestation and a decreased granulocyte signal for only preeclampsia without severe features at 12 weeks or less of gestation (Fig. 4b). Functional enrichment analysis further revealed pathways specific to genes that were only decreased for severe preeclampsia in early gestation (group 4); for example, axon guidance, nervous development and metabolism of RNA (*P* < 0.05; one-sided hypergeometric test with multiple hypothesis correction; see Methods).

### cfRNA reflects maternal organ health

We then investigated the possibility of monitoring organ health in a non-invasive﻿ manner. We focused on eight organ systems (Fig. 4b) relevant to preeclampsia presentation with consequences such as proteinuria, impaired liver function, renal insufficiency and epilepsy. We found substantial shifts in total contributions for all systems. We observed an increased astrocyte signal before 20 weeks of gestation and decreased oligodendrocytes and excitatory neurons at 23 or more weeks of gestation for all preeclampsia relative to normotensive (Fig. 4b). Although placental contributions increased over pregnancy with a peak in late gestation as expected, placental tissue and syncytiotrophoblast contributions were reduced for preeclampsia pregnancies before 20 weeks of gestation. Finally, we observed a decreased signal in hepatocyte, kidney, endothelial cell and smooth muscle signatures across gestation and an increased platelet signal before 12 weeks of gestation for preeclampsia. These tissue- and cell-type-specific changes are consistent both with common preeclampsia pathogenesis and with the specific, prominent diagnoses in our cohort (for example, thrombocytopenia, proteinuria, impaired liver function and renal insufficiency).

## Discussion

Our findings provide molecular evidence that supports the present physiological understanding of preeclampsia pathogenesis: early abnormal placentation and systemic endothelial dysfunction^6^. Early in gestation, we observe a reduced placental signal for preeclampsia, regardless of onset type or symptom severity. Concurrently, platelets and endothelial cells drive changes in cfRNA in preeclampsia samples regardless of symptom severity as compared to normotensive samples and between individuals with preeclampsia with and without severe symptoms, especially before 20 weeks of gestation. Increases in cell-type-specific cfRNA may occur﻿ in part through signalling and secretion by cells, as underscored by functional enrichment analysis. The innate and adaptive immune system also heavily contribute to cfRNA changes in preeclampsia, with marked shifts related to bone marrow, T cells, B cells, granulocytes and neutrophils—consistent with previous studies on the maternal–placental interface and preeclampsia﻿^6,36–38,39^.

Given the diversity of clinical presentations in preeclampsia, we propose a non-invasive means of monitoring a mother’s risk of specific organ damage. The cfRNA changes that we characterized here reflect dysfunction in at least five organ systems (brain, liver, kidney, muscle and bone marrow), and can in some cases further distinguish between preeclampsia with and without severe symptoms. As a molecular lens into maternal health, liquid biopsies present an opportunity both as a research and clinical tool to learn about the pathogenesis of a human disease in humans and as a predictor of maternal health. Here we have shown proof of principle that cfRNA measurements can form the basis for a robust liquid biopsy test, which predicts preeclampsia very early in gestation. If validated in controlled clinical studies of suitably large, racially and ethnically diverse populations, such tests could help to discover and manage individuals who are at risk for preeclampsia, complementing recent efforts based on clinical and laboratory data^40^. We have also shown here that cfRNA measurements reflect who is at risk for specific organ damage. Together, these results form the basis for a series of clinical tests that can be used to help to characterize and stratify the pathogenesis of preeclampsia in real time, and thereby to meet key objectives for obstetric care.

## Methods

### Clinical study design

The discovery and validation 1 cohorts were collected as part of a longitudinal, prospective study. We enrolled pregnant mothers (aged 18 years or older) receiving routine antenatal care on or before 12 weeks of gestation at Lucile Packard Children’s Hospital at Stanford University, following study review and approval by the Institutional Review Board (IRB) at Stanford University (21956). All individuals signed informed consent before enrolment. Whole-blood samples for plasma isolation were then collected at three distinct time points during their pregnancy course and once (or twice for two individuals) post-﻿partum. To split the larger Stanford cohort into Discovery and Validation 1, we first allocated samples using sequencing batches of which there were three. We allocated the sequencing batch with the most preeclampsia samples to Discovery to ensure sufficient statistical power and the second most preeclampsia samples to Validation 1. Sequencing batches themselves contained randomly allocated samples based on the individual such that all samples from the same individual were in the same batch. For the final sequencing batch, we randomly allocated individuals to either Discovery or Validation 1 such that all samples from 1 individual were part of the same group (either Discovery or Validation 1) and we maintained at least a 1–2 case to control ratio in both groups.

The validation 2 cohort was collected as part of the Global Alliance to Prevent Prematurity and Stillbirth (GAPPS) Pregnancy Biorepository at Yakima Valley Memorial Hospital, Swedish Medical Center and the University of Washington Medical Center under review and approval by Advarra IRB (CR00195799). Samples were processed and sequenced at Stanford under the same IRB as above (21956). All individuals signed informed consent before enrolment. Whole-blood samples for plasma isolation were collected at a single time point (or two time points in the case of two individuals with preeclampsia) before or at 16 weeks of gestation.

For all three cohorts﻿, we chose a case to control ratio of approximately 1–2 to increase statistical power. We also ensured that case and control groups were matched for race and ethnicity, and that all included individuals did not have chronic hypertension or gestational diabetes. No other matching or exclusion criterion were used; we performed no further sample selection prior to sample processing. Mothers were defined as having preeclampsia on the basis of current American College of Obstetrics and Gynecology (ACOG) guidelines (see below). Mothers were defined as controls if they had uncomplicated term pregnancies and either normal spontaneous vaginal or caesarean deliveries. For mothers who developed preeclampsia, all antenatal samples included in this study were collected before clinical diagnosis.

We processed samples from 88 individuals (60 normotensive, 28 with preeclampsia) in the discovery cohort, 43 individuals (34 normotensive, 9 with preeclampsia) in the validation 1 cohort and 87 individuals (61 normotensive, 26 with preeclampsia) in the validation 2 cohort. For some cohorts, only a subset of these individuals was included in the final analysis after filtering samples on the basis of pre-defined quality metrics (see ‘Sample quality filtering’, Supplementary Note 1, Supplementary Table 1)

We tested for within-cohort (normotensive versus preeclampsia) and across-cohort differences in demographic variables using a two-sided chi-squared test and ANOVA for categorical and continuous variables. respectively. We then applied Bonferroni correction and reported any differences as significant if ﻿adjusted *P* ≤ 0.05. Investigators were blinded during data collection as pregnancy outcomes were not known yet. Investigators were not blinded during data analysis as analysis methods required knowledge of outcome (that is, supervised learning).

### Definition of preeclampsia

Preeclampsia was defined per the ACOG guidelines^27^ on the basis of two diagnostic criteria: (1) new-onset hypertension developing on or after 20 weeks of gestation; and (2) new-onset proteinuria or in its absence, thrombocytopenia, impaired liver function, renal insufficiency, pulmonary oedema or cerebral or visual disturbances.

New-onset hypertension was defined when the systolic and/or diastolic blood were at least 140 or 90 mmHg, respectively, on at least two separate occasions between four hours and one week apart. Proteinuria was defined when either 300 mg protein was present within a 24-h urine collection or an individual urine sample contained a protein/creatinine ratio of 0.3 mg dl^−1^, or if these were not available, a random urine specimen had more than 1 mg protein as measured by dipstick. Thrombocytopenia, impaired liver function and renal insufficiency were defined as a platelet count of less than 100,000 per µl, liver transaminase levels two or more times higher than normal and serum creatinine level of higher than 1.1 mg dl^−1^, respectively.

Symptoms were defined as severe per the ACOG guidelines. Specifically, preeclampsia was defined as severe if any of the following symptoms were present and diagnosed as described above: new-onset hypertension with systolic and/or diastolic blood pressure of at least 160 and/or 110 mmHg, respectively, thrombocytopenia, impaired liver function, renal insufficiency, pulmonary oedema, new-onset headache unresponsive to medication and unaccounted for otherwise or visual disturbances.

Finally, a pregnant mother was considered to have early-onset preeclampsia if onset occurred before 34 weeks of gestation and late onset thereafter.

### Sample preparation

#### Plasma processing

At Lucile Packard Children’s Hospital, blood samples were collected in either EDTA-coated (368661, Becton-Dickinson) or Streck cfRNA BCT (218976, Streck) tubes at ≤12, 13–20 and ≥23 weeks of gestation, and post-partum for each participant. Within 30 min, tubes were then centrifuged at 1,600*g* for 30 min at room temperature. Plasma was transferred to 2-ml microfuge tubes and centrifuged at 13,000*g* for another 10 min in a microfuge. One-millilitre aliquots were then transferred to 2-ml Sarstedt screw cap microtubes (50809242, Thermo Fisher Scientific) and stored at −80 °C until analysis.

At GAPPS, blood samples were collected in EDTA-coated tubes at ≤16 weeks of gestation from a network of collection sites. Per standard operating procedure, tubes were then centrifuged within 2 h of collection at 2,500 rpm for 10 min at room temperature in a swinging bucket rotor. Plasma was transferred to 2-ml cryovials in at most 1-ml aliquots and stored at −80 °C until analysis. Sample volume was also recorded.

#### cfRNA isolation

In 96-sample batches, cfRNA from 1-ml plasma samples was extracted in a semi-automated manner using the Opentrons 1.0 system and Norgen Plasma/Serum Circulating and Exosomal RNA Purification 96-Well Kit (Slurry Format) (29500, Norgen). Samples were subsequently treated with Baseline-ZERO DNAse (DB0715K, Lucigen) for 20 min at 37 °C. DNAse-treated cfRNA was then cleaned and concentrated into 12 µl using Zymo RNA Clean and Concentrator-96 kits (R1080).

After cfRNA extraction from plasma samples, isolated RNA concentrations were estimated for a randomly selected 11 samples per batch using the Bioanalyzer RNA 6000 Pico Kit (5067-1513, Agilent) per the manufacturer’s instructions.

#### Sequencing library preparation

cfRNA sequencing libraries were prepared with the SMARTer Stranded Total RNAseq Kit v2 - Pico Input Mammalian Components (634419, Takara) from 4 µl of eluted cfRNA according to the manufacturer’s instructions. Samples were barcoded using the SMARTer RNA Unique Dual Index Kit – 96U Set A (634452, Takara), and then pooled in an equimolar manner and sequenced on Illumina’s NovaSeq platform (2 × 75 bp) to a mean depth of 54, 33 and 38 million reads per sample for discovery, validation 1, and validation 2 cohorts, respectively. Some samples (12, 61 and 0 for discovery, validation 1 and validation 2 cohorts) were not sequenced owing to failed library preparation.

### Bioinformatic processing

For each sample, raw sequencing reads were trimmed using Trimmomatic (v.0.36) and then mapped to the human reference genome (hg38) with STAR (v.2.7.3a). Duplicate reads were then removed by GATK’s (v.4.1.1) MarkDuplicates tool. Finally, mapped reads were sorted and quantified using htseq-count (v.0.11.1) generating a counts table (genes × samples). Read statistics were estimated using FastQC (v.0.11.8).

Across samples, the bioinformatic pipeline was managed using Snakemake (v.5.8.1). Read and tool performance statistics were aggregated across samples and steps using MultiQC (v.1.7). After sample quality and gene filtering, all gene counts were adjusted to log_2_-transformed counts per million reads (CPM) with trimmed mean of M values (TMM) normalization^41^.

### Sample quality filtering

For every sequenced sample, we estimated three quality parameters as previously described^42,43^. To estimate RNA degradation in each sample, we first counted the number of reads per exon and then annotated each exon with its corresponding gene ID and exon number using htseq-count. Using these annotations, we measured the frequency of genes for which all reads mapped exclusively to the 3′-most exon as compared to the total number of genes detected. RNA degradation for a given sample can then be approximated as the fraction of genes in which all reads mapped to the 3′-most exon. To estimate the number of reads that mapped to genes, we summed counts for all genes per sample using the counts table generated from bioinformatic processing above. To estimate DNA contamination, we quantified the ratio of reads that mapped to intronic as compared to exonic regions of the genome.

After measuring these metrics across nearly 700 samples, we empirically estimated RNA degradation and DNA contamination’s 95th percentile bound. We considered any given sample an outlier, low-quality sample if its value for at least one of these metrics was greater than or equal to the 95th percentile bound or if no reads were assigned to genes.

Once values for each metric were estimated across the entire dataset, we visualized: (1) whether low-quality samples clustered separately using hierarchical clustering (average linkage, Euclidean distance metric); and (2) whether sample quality drove variance in gene measurements using principal component analysis (PCA). These analyses were performed in Python (v.3.6) using Scikit-learn for PCA (v.0.23.2), Scipy for hierarchical clustering (v.1.5.1) and nheatmap for heat map and clustering visualization (v.0.1.4).

After confirming sample quality, 404 samples from 199 mothers (142 normotensive, 57 with preeclampsia) were included in the final analysis (Supplementary Table 1). Specifically, 209, 106 and 89 samples from 73, 39 and 87 participants (49, 32 and 61 normotensive; 24, 7 and 26 with preeclampsia) were included in discovery, validation 1 and validation 2, respectively.

### Gene filtering

We performed filtering to identify well-detected genes across the entire cohort. Specifically, we used a basic cut-off that required a given gene be detected at a level of at least 0.5 CPM in at least 75% of discovery samples after removing outlier samples. Following this step, we retain 7,160 genes for differential expression analysis.

### Differential expression analysis

Differential expression analysis was performed in R using Limma (v.3.38.3). To identify gene changes associated with preeclampsia across gestation and post-partum, we used a mixed-effects model. We performed differential expression analysis using two design matrices: (1) examine the interaction between time to preeclampsia onset or delivery for normotensive and preeclampsia symptoms (that is, preeclampsia with or without severe symptoms); and (2) examine the interaction between time to preeclampsia onset or delivery for normotensive and preeclampsia broadly. In both design matrices, we included time to preeclampsia onset or delivery for normotensive (continuous variable), whether a sample was collected post-partum (binary variable), the interaction between time and preeclampsia symptoms for (1) or preeclampsia for (2), the interaction between whether a sample is post-partum and preeclampsia symptoms for (1) and preeclampsia for (2), and 7–8 confounding factors.

In (1), we defined preeclampsia symptoms categorially using three levels—normotensive, preeclampsia without severe symptoms, preeclampsia with severe symptoms. In (2), we defined whether a sample was preeclampsia using a binary, indicator variable (0 = normotensive, 1 = preeclampsia). The 7–8 confounding variables included were maternal race (categorical variable), maternal ethnicity (binary variable), fetal sex (binary variable), maternal pre-pregnancy BMI group (categorical variable), maternal age (continuous variable, only included in design 1), and sequencing batch (categorical variable). We defined time to preeclampsia onset or delivery as the difference between gestational age at onset or delivery and gestational age at sample collection. We defined BMI group as follows: obese (BMI ≥ 30), overweight (25 ≤ BMI < 30), healthy (18.5 ≤ BMI < 25), underweight (BMI < 18.5). We chose to model time to preeclampsia onset or delivery as a continuous variable, specifically a natural cubic spline with four degrees of freedom to account for the range across which samples were collected (one to three months per collection period). We also blocked for participant identity (categorical variable), modelling it as a random effect to account for auto-correlation between samples from the same person.

Per the Limma-Voom guide, to account for sample auto-correlation over time, we ran the function voomWithQualityWeights twice. We first ran it without any blocking on participant identity, and used this base estimation to approximate sample auto-correlation on the basis of participant identity using the function duplicateCorrelation. After estimating correlation, voomWithQualityWeights was run again, this time blocking for participant identity and including the estimated auto-correlation level. A linear model was then fit for each gene using lmFit and differential expression statistics were approximated using Empirical Bayes (eBayes) methods. For comparing preeclampsia with versus without severe symptoms, we contrasted the relevant coefficients (makeContrasts) and then applied Empirical Bayes as opposed to directly after lmFit.

DEGs were then identified using the relevant design matrix coefficients with Benjamini–Hochberg multiple hypothesis correction at a significance level of 0.05. For design 1, we identified DEGs related to three comparisons: preeclampsia without severe symptoms versus normotensive (1,759 DEGs); severe preeclampsia versus normotensive (1,198 DEGs); and preeclampsia with versus without severe symptoms (503 DEGs). We find 544 genes in common for preeclampsia without and with severe symptoms versus normotensive. These 544 DEGs are examined in Fig. 2 and the related main text. For design 2, we identified DEGs related to preeclampsia versus normotensive alone (330 DEGs), which we used as the initial gene set for building a logistic regression model (see Supplementary Note 2). Finally, we removed the effect of sequencing batch on estimated logCPM values with TMM normalization for the discovery cohort using the limma-voom function, removeBatchEffect.

### log_2_-transformed fold change and CV estimation

We define log_2_-transformed fold-change (﻿log_2_(FC)) as the difference between the median gene level (logCPM; see ‘Bioinformatic processing’) between preeclampsia and normotensive samples for a given sample collection period (that is, ≤12, 13–20 and ≥23 weeks of gestation, or post-partum). In the case in which a given person had multiple samples included into a specific collection period, we only used the values associated with the first collected sample to avoid artificially reducing within-group (preeclampsia or normotensive) variance due to auto-correlation among samples from the same person.

We then quantified the relative dispersion around the estimated log﻿_2_(FC) for each gene using an approximation for CV. Specifically, we consider CV to be the ratio between an error bound, ∂, and the estimated log﻿_2_(FC). We defined the error bound, ∂, as the one-sided error bound associated with the lower (or upper in the case of negative ﻿log_2_(FC) values) 95% CI as estimated by bootstrapping. This definition of ∂ as a one-sided bound that approaches 0 (equivalent to no FC) allowed us to explore how confident we could be in an estimated ﻿log_2_(FC). For instance, a CV = 1 would indicate that at the boundary of proposed values, the log﻿_2_(FC) for a given gene becomes effectively 0. Similarly, a CV > 1 would indicate even less confidence in a proposed average ﻿log_2_(FC) and indicate that at the boundary, the estimated ﻿log_2_(FC) changes signs (that is, a negative ﻿log_2_(FC) becomes a positive one or vice versa).

### Hierarchical clustering analysis

For each sample collection period, hierarchical clustering was performed using DEGs with an |﻿log_2_(FC)| ≥ 1 and CV < 0.5 or 0.4 in the case of the 13–20 weeks of gestation time point so that the number of genes used did not exceed the number of samples. For each gene that passed these thresholds, we calculated a *z*-score across all samples (at most 1 per individual, the earliest collected sample in a given group) in each sample collection period using the function StandardScaler in Scikit-learn (v.0.23.2), Average linkage hierarchical clustering with a Euclidean distance metric was then performed for both rows (gene *z*-scores) and columns (samples in same collection group) for a given matrix in Python using Scipy (v.1.5.1). Clustering and corresponding heat maps were visualized using nheatmap (v.0.1.4).

### Longitudinal dynamics analysis

To group gene changes by longitudinal behaviour, we performed *k*-means clustering on a matrix in which each row was a DEG and each column was the estimated ﻿log_2_(FC) for a given sample collection period (*N* genes × 4 time points). We measured the sum of squared distances for every ‘*k*’ between 1 and 16 (4^2^), in which 16 represents the maximum possible *k* (4 time points with 2 possibilities each, ﻿log_2_(FC) > 0 or ﻿log_2_(FC) < 0). We then identified the optimal *k* clusters by using the well-established elbow method to identify the smallest ‘*k*’ that best explained the data, visually described as the elbow (or knee) of a convex plot like that for the sum of squared distances versus *k* (Extended Data Fig. 4a, c). To do so, we either visually inspected and identified the elbow (Fig. 2d) or used the function KneeLocator as implemented in the package Kneed (v.0.7.0) (Fig. 4a). We used visual inspection for Fig. 2d as we observed that given two *k*-values (for example, *k* = 2, 3) with a similar sum of squared distances, KneeLocator would choose the larger value, whereas we preferred a simpler model. Having identified the optimal number of clusters, *k*, we labelled every DEG with its assigned cluster and visualized average behaviour (median) and the 95% CI (bootstrapped using 1,000 iterations) per cluster using Seaborn line plot (v.0.10.0).

To confirm that the identified patterns were not spurious (that is, an artifact of the *k*-means clustering algorithm), we permuted the data columns (﻿log_2_(FC) per time point) for each gene thereby scrambling any time-related structure while preserving its overall distribution. We then visualized the result using Seaborn line plot as described above. After permutation, we observed no longitudinal patterns, which were instead replaced by nearly flat, uninformative trends (Extended Data Fig. 4b, d).

### Correlation analysis

To verify DEGs identified in the discovery cohort, we compared ﻿log_2_(FC) values for the discovery cohort as compared to both validation 1 and validation 2 cohorts. We calculated the percentage of genes for which the ﻿log_2_(FC) had the same sign across cohorts (that is, both positive or both negative) and the Spearman correlation using the function scipy.stats.spearmanr. We did not calculate log﻿_2_(FC) values for DEGs at ≤12 weeks of gestation in validation 1 because of small sample numbers (3 preeclampsia samples before 12 weeks).

We also sought to compare whether symptom severity (without or with severe preeclampsia) correlated with ﻿log_2_(FC) magnitude for 544 DEGs identified as common to all preeclampsia in design 1. To do so, we calculated the slope of a best-fit line in which *x* and *y* were defined as ﻿log_2_(FC) values for preeclampsia without (*x*) and with (*y*) severe features versus normotensive. We would expect a slope > 1 and < 1 if log﻿_2_(FC) magnitudes for preeclampsia with as compared to without severe symptoms were larger or smaller on average, respectively. Similarly, a slope = 1 would reflect that symptom severity did not correlate with ﻿log_2_(FC) magnitude for the 544 DEGs tested.

Finally, to confirm that the identified correlations were significant, we permuted the data columns (log﻿_2_(FC) per cohort) for each gene, thereby scrambling any structure while preserving its overall distribution. We then calculated the same statistics. After permutation, we observe about 50–55% log﻿_2_(FC) agreement, as expected at random, a Spearman correlation of 0 and a slope of 0.

### Defining cell-type- and tissue-specific gene profiles

Cell-type gene profiles were identified as previously described^26^ and briefly summarized below. We also briefly describe an adapted, similar method to derive tissue gene profiles.

On the tissue level, for genes and tissues (and some blood and immune cell types) measured in the HPA (v.19)^35^, we calculated a Gini coefficient per gene as a measure of tissue specificity. As a measure of inequality, Gini coefficient values closer to 1 represent genes that are tissue-specific. We defined a given gene *Y* as specific to tissue *X* included in the HPA if Gini(*Y*) ≥ 0.6 and max expression for *Y* is in tissue *X*. The aforementioned method can identify genes that are expressed in several tissues (for example, group-enriched) as opposed to only one. Specifically, it is possible to have a gene *Y* where Gini(*Y*) ≥ 0.6 and the gene is expressed in more than 1 tissue (for example, enrichment in placenta and muscle). To this end, when tracking a single cell type or tissue’s trajectory over gestation (for example, Fig. 4b), where the specificity of a given gene profile is especially important, we imposed a further constraint to ensure that any gene signal only reflects the named tissue (for example, any gene named placenta-specific is specific to the placenta alone). Specifically, we required that genes be annotated by HPA as ‘Tissue-enriched’ or ‘Tissue-enhanced’ and term this reference ‘HPA strict’.

On the cell-type level, we identified cell-type-specific gene profiles using both Tabula Sapiens v.1.0 (TSP) and individual cell atlases. We used individual cell atlases to identify gene profiles for cell types from missing tissues in TSP (for example, placenta, brain) or tissues that are known to be important in preeclampsia with additional annotations in individual single-cell atlases (for example, liver, kidney). First, for genes and cell types measured in TSP, we defined a given gene *Y* as specific to cell type *X* included in TSP if Gini(*Y*) ≥ 0.8 and max mean expression for *Y* is in cell type *X*. We﻿ combined subtype annotations for neutrophil and endothelial cells into single parent categories called ‘neutrophil’ and ‘endothelial cell’ respectively; as subtype annotations were based on manifold clustering, it was unclear whether they were truly distinct enough to be distinguished at a whole-body level for our purposes. Next, for genes and cell types described in individual tissue single-cell atlases, we required that a gene be differentially expressed in the specific single-cell atlases and tissue-specific per the HPA (Gini ≥ 0.6). The following single-cell atlases were used for each organ: (1) placenta^44,45^; (2) liver^46^; (3) kidney^47^; (4) heart^48^; and (5) brain^49^.

We then created an augmented reference, which we term TSP+. For TSP+, we took the union of TSP and individual atlas gene annotations. A small number of genes had conflicting double annotations in TSP as compared to at most one individual tissue single-cell atlas. In these rare instances, which most often occurred for genes related to cell types missing in TSP (for example, placental or brain cell types), we used the individual single-cell atlas label.

### Determining cell type and tissue of origin

We determined whether a given cell type or tissue was enriched in preeclampsia by comparing preeclampsia DEGs with cell-type and tissue gene profiles using a one-sided hypergeometric test. For every tissue (HPA) or cell type (TSP+) with at least two DEGs specific to it, we performed the following. First, we defined a hypergeometric distribution (scipy.stats.hypergeom, (v.1.5.1)) with the following parameters, where category refers to tissue when using HPA and cell type when using TSP+: *M* = number of genes specific to any category; *n* = number of genes specific to this category; *N* = number of DEGs in this *k*-means longitudinal cluster specific to this category. Next, we estimated a *P* value using the survival function (1 − cumulative distribution function (CDF)) for the specified distribution. Specifically, a *P* value is defined as the cumulative probability, *P*(*X* > (*n_*DEGs_specific_to_this_category − 1)), that the distribution takes a value greater than the number of DEGs specific to this category − 1. Finally, once we estimated a *P* value for every cell type (TSP+) or tissue (HPA) identified in each DEG *k*-means longitudinal cluster, we adjusted for multiple hypotheses using Benjamini–Hochberg with a significance threshold of 0.05.

### Defining relative signature score per cell type or tissue

We define a signature score as the sum of logCPM values over all genes in a given tissue or cell-type gene profile^26^. We required that a cell-type or tissue gene profile have at least five specified genes to be considered for signature scoring in cfRNA. Genes were defined as specific to a given tissue on the basis of the reference HPA strict, and to a given cell type on the basis of the reference TSP+ (see ‘Defining cell-type- and tissue-specific gene profiles’ for details).

To account for our previous observation that baseline cfRNA levels vary between individuals—the consequence of biological and technical (for example, sample processing) factors, we chose to calculate relative as opposed to absolute signature scores. For each individual whose post-partum sample passed sample quality control (QC) (see ‘Sample quality filtering’ for details), we estimated a relative signature score, which was defined as the difference between the signature score at a given gestational time point and the post-partum sample. For both the discovery cohort and the validation 1 cohort, 49 normotensive individuals and 24 individuals with preeclampsia had a post-partum sample that survived sample QC. After normalization, all samples at post-partum had a similar baseline (0). We note that one can define a relative signature score based on any sampled time point for a given person. We chose the post-partum sample because we were interested in tracking maternal organ health over gestation.

Finally, we scaled (that is, *z*-score) the relative signature scores for a given cell type or tissue by dividing by the interquartile range, a robust alternative to standard deviation, using the sklearn.preprocessing class, RobustScaler. This accounted for differing gene profile lengths and gene expression levels, and allowed us to compare both different cell-type and tissue contributions and case groups per cell type or tissue.

Having defined a relative signature score per cell type and tissue, we visualized average behaviour (median) and the 75% CI, a non-parametric estimation of standard deviation, (bootstrapped relative signature score per case group and time point using 1,000 iterations) using Seaborn line plot (v.0.10.0).

### Functional enrichment analysis

Functional enrichment analysis was performed using the tool GProfiler (v.1.0.0) for the following data sources: Gene Ontology: biological processes and cellular compartments (GO:BP, GO:CC, released 1 May 2021, 2021-05-01), Reactome (REAC, released 7 May 2021, 2021-05-07) and Kyoto Encyclopedia of Genes and Genomes (KEGG FTP, released 3 May 2021, 2021-05-03). To identify GO terms, we excluded electronic GO annotations (IEA) and used a custom background of only the 7,160 genes that were included in the differential expression analysis. We then performed the recommended multiple hypothesis correction (g:SCS) with an experiment-wide significance threshold of *a* = 0.05 (ref. ^50^).

### Logistic regression feature selection and training

To build a robust classifier that can identify mothers at risk of preeclampsia at or before 16 weeks of gestation, we first pre-selected features using the set of 330 DEGs when contrasting preeclampsia versus normotensive (see design 2 in ‘Differential expression analysis’ and Supplementary Note 2) as a starting point.

We normalized gene measurements using a series of steps. First, to correct for batch effect, in which we define batch as a set of samples processed at the same time by a distinct group (for example, discovery cohort = batch, Del Vecchio cohort = batch), we centred the data by subtracting the median logCPM per gene for a given cohort. Next, we scaled gene values for each cohort using its corresponding interquartile range in the discovery cohort. Finally, to account for sampling differences across samples, we used an approach similar to when analysing quantitative PCR with reverse transcription (RT–qPCR) data, and normalized data using multiple internal control (that is, housekeeping) genes. On a per-sample basis, we subtracted the median, normalized logCPM value (centred and scaled) for all internal control genes, which we define as 66 genes for which the measured value did not change across preeclampsia versus normotensive comparisons (all genes with adjusted *P* > 0.99 for preeclampsia versus normotensive; design 2). When calculating the median value for all internal control genes, we excluded any 0 logCPM values as these are likely to have been the consequence of technical dropout.

Model training then used the discovery cohort alone split into 80% for hyperparameter tuning and 20% for model selection and consisted of two stages—further feature pre-selection based on two metrics followed by the construction of a logistic regression model with an elastic net penalty. Using a split discovery cohort for training mitigated overfitting even though all discovery samples were used for differential expression, which defined the initial feature set.

For feature pre-selection, we calculated logFC values using the 80% discovery split for all 330 genes for preeclampsia versus normotensive. We focused on 2 practical metrics measured across the 80% split of discovery samples collected on or before 16 weeks of gestation: gene change size (|﻿log_2_(FC)|) and gene change stability (CV). All model hyperparameters were then tuned using AUROC as the outcome metric and fivefold cross validation. Next, we selected the best model including tuned feature pre-selection cut-offs again using AUROC. Specifically, we calculated an AUROC score for both the 80% and the 20% discovery splits separately, and the selected model achieved the best score on both splits.

Finally, we tuned the probability threshold, *P*, at which a sample is labelled as at risk of preeclampsia if *P*(PE) ≥ *P* using the entire discovery cohort. To do so, we constructed a receiver operator characteristic curve (ROC) and calculated the false positive rate (FPR) and true positive rate (TPR) at different thresholds, *P_i*. We identified the threshold, *P_i*, at which FPR = 10%, and round to the nearest 5 (for example, 0.37 would become 0.35). This yielded a tuned threshold of *P* = 0.35. All classifications as negative or positive were then made based on this threshold.

To understand the importance of each gene feature, we trained a separate logistic regression model for a subset of all possible feature subsets (307 combinations out of a total of 262,143 for 1–17 genes). No feature pre-selection was performed for this sub-analysis. All model hyperparameters were tuned as previously described. We defined a gene subset as weakly predictive if the model yielded an AUROC > 0.5 on the test set (validation 2).

In all cases, performance metrics were assessed as described below (see next section) and used Scikit-learn (v.0.23.2).

### Performance metric analysis

Model performance was assessed using several statistics including sensitivity, specificity, positive predictive value (PPV), negative predictive value (NPV) and AUROC. Given a 2 × 2 confusion matrix in which rows 1and 2 represent true negatives and positives and columns 1 and 2 represent negative and positive predictions, respectively, we can define the values in row 1, column 1 as true negatives (TN), row 1, column 2 as false positives (FP), row 2, column 1 as false negatives (FN), and row 2, column 2 as true positives (TP). We can then define the following proportions: (1) sensitivity = TP/(TP + FN); (2) specificity = TN/(TN + FP); (3) PPV = TP/(TP + FP); (4) NPV = TN/(TN + FN). For each proportion, we calculated 90% CIs using Jeffrey’s interval^51^ and the function, proportion_confint, from statsmodels.stats.proportion. We also approximated AUC and its corresponding 90% CI using the Scikit-learn function, roc_auc_score, and the binormal approximation, respectively.

### Statistical analyses

All *P* values reported herein were calculated using the non-parametric Mann–Whitney rank test unless otherwise stated. One-sided tests were performed where required based on the hypothesis tested.

### Reporting summary

Further information on research design is available in the Nature Research Reporting Summary linked to this paper.

## Online content

Any methods, additional references, Nature Research reporting summaries, source data, extended data, supplementary information, acknowledgements, peer review information; details of author contributions and competing interests; and statements of data and code availability are available at 10.1038/s41586-022-04410-z.

### Supplementary information

Supplementary Information This file contains Supplementary Notes 1 and 2; Supplementary Tables 1, 2, 5 and 6 and legends for Supplementary Tables 3 and 4.

Reporting Summary

Supplementary Table 3 See Supplementary Information file for description.

Supplementary Table 4 See Supplementary Information file for description.

## Figures and Tables

**Extended Data Table 1:**
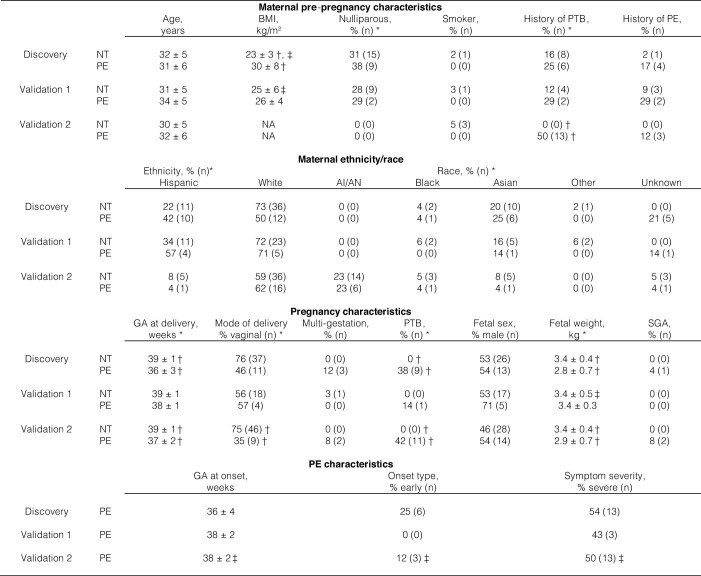
Participant, pregnancy and preeclampsia characteristics across both discovery and validation cohorts

Maternal age and BMI, gestational age (GA) at delivery, fetal weight and GA at preeclampsia onset are reported as mean ± s.d. All other values are reported as percentages with the corresponding count in parentheses. Small for GA (SGA) was defined as an infant with a birthweight below the 10th centile for their GA at delivery. Pre-pregnancy BMI was not available for individuals in the validation 2 cohort. See Supplementary Table 2 for exact *P* values. AI/AN, American Indians and Alaska Natives.

*P ≤ 0.05, two-sided chi-squared (categorical) or ANOVA (continuous) test comparing all cohorts with Bonferroni correction.

†*P* ≤ 0.05, two-sided chi-squared (categorical) or ANOVA (continuous) test comparing preeclampsia and normotensive within each cohort with Bonferroni correction.

‡ denotes that missing values were omitted from reported values for a given feature.

**Extended Data Table 2:**
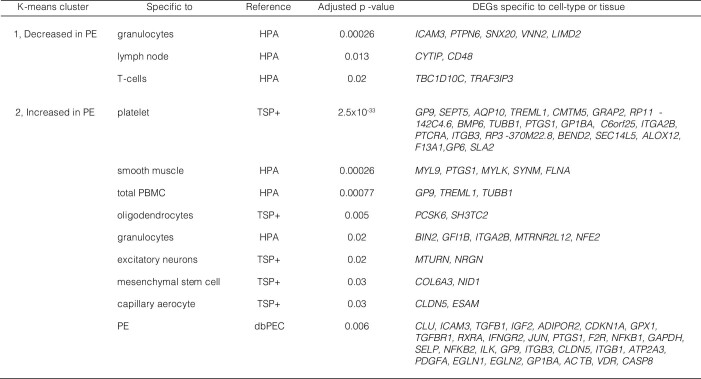
Tissue, cell types, and genes previously implicated in preeclampsia enriched in 544 DEGs identified when comparing preeclampsia with or without severe features and normotensive pregnancies

For every significantly enriched tissue or cell type (*P* ≤ 0.05; one-sided hypergeometric test with Benjamini–Hochberg correction), assigned *k*-means cluster (Fig. 2d), reference and adjusted *P* values are reported from left to right. The rightmost column lists the gene names for all DEGs that were labelled as specific to a given cell type or tissue. The last row lists gene set enrichment with dbPEC, a preeclampsia-specific database of genes.

**Extended Data Table 3﻿:**
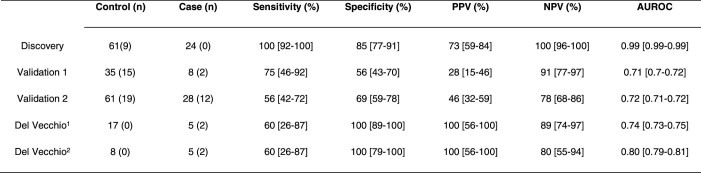
Preeclampsia prediction performance metrics for samples collected early in gestation (5–16 weeks)

Control and case sample numbers are reported as the total sample number and, in parentheses, the number of samples misclassified. All other statistics including sensitivity specificity, PPV, NPV and AUROC are reported as the estimated percentage followed by the 90% CI in square brackets. In Del Vecchio^1^, the control group is defined as samples from any pregnant mother who did not develop preeclampsia, including those with other underlying or pregnancy-related complications such as chronic hypertension or gestational diabetes, respectively. In Del Vecchio^2^, the control group is defined as samples strictly from normotensive pregnant mothers who did not experience complications.

**Extended Data Table 4:**
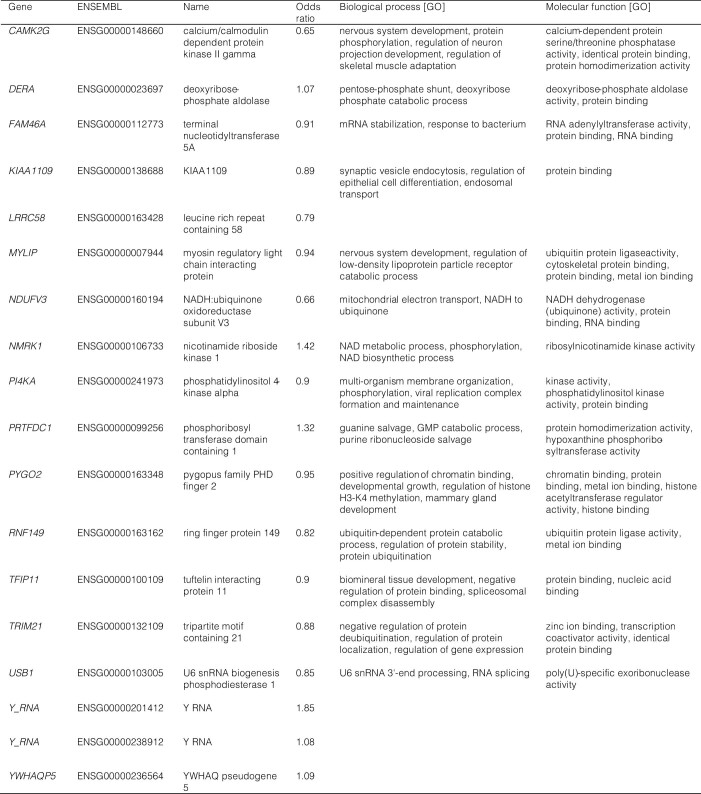
Preeclampsia prediction relies on the cfRNA levels of 18 genes

For every gene, symbol, ENSEMBL ID, full name, odds ratio (OR) based on the logistic regression coefficient and, if available, a subset of GO biological processes and molecular functions (if available) are reported from left to right.

**Extended Data Table 5:**
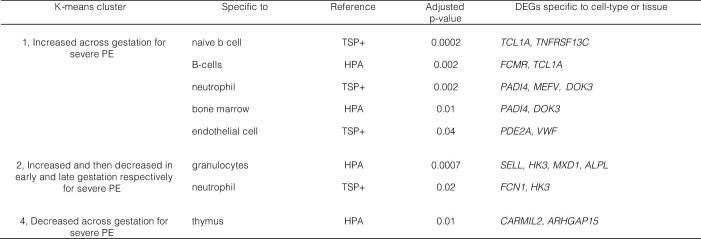
Tissue and cell types enriched in 503 DEGs identified when comparing preeclampsia with as compared to without severe features

For every significantly enriched tissue or cell type (*P* ≤ 0.05; one-sided hypergeometric test with Benjamini–Hochberg correction), assigned *k*-means cluster (Fig. 4a), reference and adjusted *P* values are reported from left to right. The rightmost column lists the gene names for all DEGs that were labelled as specific to a given cell type or tissue.

## Data Availability

Raw and processed sequencing data (BioProject PRJNA792450) are available through the Sequence Read Archive (SRA, SRP352519) and the Gene Expression Omnibus (GEO, GSE192902), respectively. Data were mapped using the human reference genome (hg38) and annotated using ENSEMBL v.82. We used publicly available data from the HPA (v.19; https://v19.proteinatlas.org/); TSP (v.1.0; https://tabula-sapiens-portal.ds.czbiohub.org/); Gene Ontology: biological processes and cellular compartments (GO:BP, GO:CC, released 1 May 2021, 2021-05-01); Reactome (REAC, released 7 May 2021, 2021-05-07); Kyoto Encyclopedia of Genes and Genomes (KEGG, released 3 May 2021, 2021-05-03); and several previous publications^44–49^.

## Code availability

All computational analyses were performed using Python 3.6 or R 3.5, and are available on Github at https://github.com/miramou/pe_cfrna.

## References

[1] Blencowe H (2013). Born too soon: the global epidemiology of 15 million preterm births. Reprod. Health.

[2] Stevens W (2017). Short-term costs of preeclampsia to the United States health care system. Am. J. Obstet. Gynecol..

[3] Basso O (2006). Trends in fetal and infant survival following preeclampsia. JAMA.

[4] Beam AL (2020). Estimates of healthcare spending for preterm and low-birthweight infants in a commercially insured population: 2008–2016. J. Perinatol..

[5] Say L (2014). Global causes of maternal death: a WHO systematic analysis. Lancet Glob. Health.

[6] Phipps EA, Thadhani R, Benzing T, Karumanchi SA (2019). Pre-eclampsia: pathogenesis, novel diagnostics and therapies. Nat. Rev. Nephrol..

[7] Pennington KA, Schlitt JM, Jackson DL, Schulz LC, Schust DJ (2012). Preeclampsia: multiple approaches for a multifactorial disease. Dis. Model. Mech..

[8] Steegers EAP, von Dadelszen P, Duvekot JJ, Pijnenborg R (2010). Pre-eclampsia. Lancet.

[9] Burton GJ, Redman CW, Roberts JM, Moffett A (2019). Pre-eclampsia: pathophysiology and clinical implications. BMJ.

[10] McCarthy FP, Ryan RM, Chappell LC (2018). Prospective biomarkers in preterm preeclampsia: a review. Pregnancy Hypertens..

[11] Henderson JT, Vesco KK, Senger CA, Thomas RG, Redmond N (2021). Aspirin use to prevent preeclampsia and related morbidity and mortality: updated evidence report and systematic review for the US preventive services task force. JAMA.

[12] Behrman, R. E. & Butler, A. S. in *Preterm Birth: Causes, Consequences, and Prevention* (eds Berhman, R. E. & Butler, A. S.) Ch. 12 (National Academic Press, 2007). 20669423

[13] Petersen EE (2019). Vital signs: pregnancy-related deaths, United States, 2011–2015, and strategies for prevention, 13 states, 2013–2017. MMWR Morb. Mortal. Wkly Rep..

[14] The American College of Obstetricians and Gynecologists. (2013). Hypertension in pregnancy. Report of the American College of Obstetricians and Gynecologists’ Task Force on Hypertension in Pregnancy. Obstet. Gynecol..

[15] Goel A (2015). Epidemiology and mechanisms of de novo and persistent hypertension in the postpartum period. Circulation.

[16] Whitehead CL, Walker SP, Tong S (2016). Measuring circulating placental RNAs to non-invasively assess the placental transcriptome and to predict pregnancy complications. Prenat. Diagn..

[17] Munchel S (2020). Circulating transcripts in maternal blood reflect a molecular signature of early-onset preeclampsia. Sci. Transl. Med..

[18] Tsang JCH (2017). Integrative single-cell and cell-free plasma RNA transcriptomics elucidates placental cellular dynamics. Proc. Natl Acad. Sci. USA.

[19] Del Vecchio G (2021). Cell-free DNA methylation and transcriptomic signature prediction of pregnancies with adverse outcomes. Epigenetics.

[20] von Dadelszen P, Magee LA, Roberts JM (2003). Subclassification of preeclampsia. Hypertens. Pregnancy.

[21] Raymond D, Peterson E (2011). A critical review of early-onset and late-onset preeclampsia. Obstet. Gynecol. Surv..

[22] Chaiworapongsa T (2013). Differences and similarities in the transcriptional profile of peripheral whole blood in early and late-onset preeclampsia: insights into the molecular basis of the phenotype of preeclampsiaa. J. Perinat. Med..

[23] Leavey K (2016). Unsupervised placental gene expression profiling identifies clinically relevant subclasses of human preeclampsia. Hypertension.

[24] Benton SJ, Leavey K, Grynspan D, Cox BJ, Bainbridge SA (2018). The clinical heterogeneity of preeclampsia is related to both placental gene expression and placental histopathology. Am. J. Obstet. Gynecol..

[25] Koh W (2014). Noninvasive in vivo monitoring of tissue-specific global gene expression in humans. Proc. Natl Acad. Sci. USA.

[26] Vorperian, S. K., Moufarrej, M. N., Tabula Sapiens Consortium & Quake, S. R. Cell types of origin in the cell free transcriptome in human health and disease. Preprint at *bioRxiv*10.1101/2021.05.05.441859 (2021).

[27] The American College of Obstetricians and Gynecologists. (2020). Gestational hypertension and preeclampsia: ACOG practice bulletin, number 222. Obstet. Gynecol..

[28] Uzun A, Triche EW, Schuster J, Dewan AT, Padbury JF (2016). dbPEC: a comprehensive literature-based database for preeclampsia related genes and phenotypes. Database.

[29] Gillich A (2020). Capillary cell-type specialization in the alveolus. Nature.

[30] Harris LK, Crocker IP, Baker PN, Aplin JD, Westwood M (2011). IGF2 actions on trophoblast in human placenta are regulated by the insulin-like growth factor 2 receptor, which can function as both a signaling and clearance receptor. Biol. Reprod..

[31] Robinson NJ, Baker PN, Jones CJP, Aplin JD (2007). A role for tissue transglutaminase in stabilization of membrane-cytoskeletal particles shed from the human placenta. Biol. Reprod..

[32] Arck PC, Hecher K (2013). Fetomaternal immune cross-talk and its consequences for maternal and offspring’s health. Nat. Med..

[33] Weissgerber TL, Mudd LM (2015). Preeclampsia and diabetes. Curr. Diab. Rep..

[34] Sibai BM (2011). The impact of prior preeclampsia on the risk of superimposed preeclampsia and other adverse pregnancy outcomes in patients with chronic hypertension. Am. J. Obstet. Gynecol..

[35] Uhlen M (2019). A genome-wide transcriptomic analysis of protein-coding genes in human blood cells. Science.

[36] Han X (2019). Differential dynamics of the maternal immune system in healthy pregnancy and preeclampsia. Front. Immunol..

[37] Ander SE, Diamond MS, Coyne CB (2019). Immune responses at the maternal–fetal interface. Sci. Immunol..

[38] Szarka A, Rigó J, Lázár L, Beko G, Molvarec A (2010). Circulating cytokines, chemokines and adhesion molecules in normal pregnancy and preeclampsia determined by multiplex suspension array. BMC Immunol..

[39] Ibarra, A. et al. Non-invasive characterization of human bone marrow stimulation and reconstitution by cell-free messenger RNA sequencing. *Nat. Commun.* **11**, 400 (2020). 10.1038/s41467-019-14253-4 PMC6972916 31964864

[40] Marić I (2020). Early prediction of preeclampsia via machine learning. Am. J. Obstet. Gynecol MFM.

[41] Robinson MD, Oshlack A (2010). A scaling normalization method for differential expression analysis of RNA-seq data. Genome Biol..

[42] Pan, W. *Development of Diagnostic Methods Using Cell-free Nucleic Acids* (Stanford University, 2016).

[43] Moufarrej MN, Wong RJ, Shaw GM, Stevenson DK, Quake SR (2020). Investigating pregnancy and its complications using circulating cell-free RNA in women’s blood during gestation. Front. Pediatr..

[44] Vento-Tormo R (2018). Single-cell reconstruction of the early maternal–fetal interface in humans. Nature.

[45] Suryawanshi H (2018). A single-cell survey of the human first-trimester placenta and decidua. Sci. Adv..

[46] Aizarani N (2019). A human liver cell atlas reveals heterogeneity and epithelial progenitors. Nature.

[47] Stewart BJ (2019). Spatiotemporal immune zonation of the human kidney. Science.

[48] Litviňuková M (2020). Cells of the adult human heart. Nature.

[49] Mathys H (2019). Single-cell transcriptomic analysis of Alzheimer’s disease. Nature.

[50] Raudvere U (2019). g:Profiler: a web server for functional enrichment analysis and conversions of gene lists (2019 update). Nucleic Acids Res..

[51] Brown LD, Cai TT, DasGupta A (2001). Interval estimation for a binomial proportion. Stat. Sci..

