# Supplementary material for: Early prediction of preeclampsia in pregnancy with cell-free RNA
Source: Nature. 2022 Feb 9;602(7898):689–94. doi: 10.1038/s41586-022-04410-z (PMC8971130; doi:10.1038/s41586-022-04410-z)
Supplement: Supplementary file 1 — This file contains Supplementary Notes 1 and 2; Supplementary Tables 1, 2, 5 and 6 and legends for Supplementary Tables 3 and 4. [file 41586_2022_4410_MOESM1_ESM.pdf]

nature portfolio

https://doi.org/10.1038/s41586-022-04410-z

Supplementary information

Early prediction of preeclampsia in pregnancy with cell-free RNA

In the format provided by the
authors and unedited

Supplementary Notes

Supplementary note 1: Establishing quality metrics to identify sample outliers

Because cfRNA measurements can be noisy [17], we have previously developed and reported on quality metrics that can flag sequenced cfRNA samples with poor quality [41,42]. Specifically, these metrics aim to quantify unusually high levels of RNA degradation and/or DNA contamination by comparing a given sample's value for any of these metrics with what we expect empirically. We defined reasonable expected values for each metric based on the 95th percentile for ~700 previously sequenced samples across 3 cohorts.

We found that samples with outlier values for at least one of these metrics both clustered separately and served as leverage points in PCA (Extended Data Fig. 1a-c). To avoid introducing unwanted bias, we removed these low-quality samples from any further analysis. After removing outlier samples, we reran PCA and noticed that some samples continued to serve as leverage points. We suspected that this may be due to genes that were poorly detected and consequently performed further filtering to identify well-detected genes across the entire cohort. Specifically, we used a basic cutoff that required a given gene be detected at a level of at least 0.5 CPM reads in at least 75% of samples after removing outlier samples. Following this step, we retain 7,160 genes for analysis. Upon inspection, we find that visualization using PCA is no longer driven by leverage points.

Supplementary note 2: Selecting an initial feature set for machine learning

We first explored whether a common gene set could describe PE with or without severe features. We observed that we could separate PE from NT samples (Fig. 2) irrespective of symptom severity and that PE with or without severe features as compared to NT had on average the same log FC (Extended Data Fig. 2e). With this in mind, we reran differential expression to identify a core set of genes that can distinguish PE (as a binary case group) from NT (See Design 2 in methods section "Differential expression analysis" for more details). This identified 330 genes that we used as an initial feature set for machine learning.

Supplementary Tables

Supplementary Table 1. Number of subjects with a given number of samples that passed QC for Discovery and Validation 1 cohorts.

	Number of samples				
	1	2	3	4	5
Discovery	5	19	31	17	1
Validation 1	7	8	14	9	1

Supplementary Table 2. Exact adjusted p-values reported for within and between cohort comparisons in Extended Data Table 1. We were not able to calculate a few p-values either because the data all belonged to one group (e.g., PE onset type for Validation 1) or data were unavailable (e.g., BMI for Validation 2). P-values were calculated using either two-sided chi-squared (categorical) or ANOVA (continuous) test with Bonferroni correction and contrasted PE and NT within each cohort (Discovery, Validation 1, 2) or between all cohorts (Between cohorts). NA; Not Applicable, NC; Not calculated.

		Discovery	Validation 1	Validation 2	Between cohorts
Maternal pre-pregnancy characteristics	Age	1	1	1	1
	BMI	0.03	1	NC	0.12
	Nulliparous	1	1	NC	4.5×10^{-5}
	Smoker	1	1	1	1
	History of PTB	1	1	7×10^{-7}	2.19×10^{-5}
	History of PE	1	1	1	0.08
Maternal ethnicity/race	Ethnicity	1	1	1	0.002
	Race	0.75	1	1	0.004
Pregnancy characteristics	GA at delivery	3×10^{-6}	1	8×10^{-9}	2.1×10^{-15}
	Mode of delivery	1	1	0.035	0.02
	Multi-gestation	1	1	1	0.43
	PTB	0.001	1	1.75×10^{-5}	5×10^{-11}
	Fetal sex	1	1	1	1
	Fetal weight	9.5×10^{-5}	1	0.01	9.94×10^{-7}
	SGA	1	NC	1	1
PE characteristics	GA at onset	NA	NA	NA	1
	Onset type	1	NC	1	1
	Symptom severity	1	1	1	1

Supplementary Table 3. Table of 89 differentially expressed genes included in Fig. 2b, Extended Data Fig. 2b heatmaps. For every gene, the symbol, ENSEMBL ID, sample collection groups for which the gene passed cutoff thresholds, full name, ENSEMBL gene type, and a subset of GO biological processes and molecular functions are reported.

Supplementary Table 4. Table of all significant GO terms related to 544 DEGS for PE with or without severe features as compared with NT ($p \leq 0.05$; one-sided hypergeometric test with multiple hypothesis correction, see Methods).

Supplementary Table 5. Exact adjusted p-values for cfRNA gene trends reported in Fig. 3b. Univariate analysis confirmed that 9 gene trends (i.e., decreased or increased gene levels in PE) observed in Discovery are upheld in Validation 2 (one-sided Mann-Whitney rank test with Benjamini-Hochberg correction).

Gene	ENSEMBL	Discovery	Validation 1	Validation 2	Del Vecchio
		Adjusted p-value			
CAMK2G	ENSG00000148660	0.0021	0.48	0.65	0.71
DERA	ENSG00000023697	0.0018	0.73	0.0008	0.69
FAM46A	ENSG00000112773	0.0018	0.47	0.008	0.69
KIAA1109	ENSG00000138688	0.037	0.48	0.53	0.31
LRRC58	ENSG00000163428	0.007	0.15	0.006	0.69
MYLIP	ENSG00000007944	0.0113	0.28	0.06	0.69
NDUFV3	ENSG00000160194	0.0001	0.63	0.008	0.69
NMRK1	ENSG00000106733	0.0083	0.48	0.006	0.69
PI4KA	ENSG00000241973	0.2217	0.98	0.01	0.69
PRTFDC1	ENSG00000099256	0.0002	0.48	0.02	0.63
PYGO2	ENSG00000163348	0.016	0.71	0.12	0.69
RNF149	ENSG00000163162	0.0064	0.61	0.25	0.69
TFIP11	ENSG00000100109	0.0018	0.71	0.015	0.63
TRIM21	ENSG00000132109	0.0001	0.39	0.008	0.69
USB1	ENSG00000103005	0.0046	0.48	0.12	0.68
YWHAQP5	ENSG00000236564	0.0044	0.73	0.84	0.65
Y_RNA	ENSG00000201412	0.0002	0.15	0.22	0.62
Y_RNA	ENSG00000238912	0.0002	0.25	0.06	0.69

Supplementary Table 6. Logistic regression models trained on some subsets of 1–18 genes of the initial 18 genes can predict future PE onset with nearly equivalent performance metrics. The associated performance metrics for each data split and some high-performing gene subsets is reported including sensitivity (Sens), specificity (Spec), PPV, NPV, and AUROC, which are reported as the estimated percentage. Only a few, illustrative examples are shown.

Subset	Size	PPV	NPV	Sens	Spec	AUROC
				Validation 2		
DERA	1	48	84	75	62	0.76
TRIM21, PI4KA	2	44	76	54	69	0.71
TRIM21, Y_RNA, DERA	3	50	78	54	75	0.77
TRIM21, USB1, PYGO2, DERA	4	50	73	32	85	0.71
LRRC58, KIAA1109, MYLIP, USB1, NMRK1	5	48	78	54	74	0.70
Y_RNA, NDUFV3, KIAA1109, MYLIP, USB1, RNF149, PRTFDC1, PI4KA, NMRK1, YWHAQP5, Y_RNA	11	67	72	21	95	0.75
