# Supplementary material for: Early prediction of preeclampsia in pregnancy with cell-free RNA
Source: Nature. 2022 Feb 9;602(7898):689–94. doi: 10.1038/s41586-022-04410-z (PMC8971130; doi:10.1038/s41586-022-04410-z)
Supplement: Supplementary file 2 — Reporting Summary [file 41586_2022_4410_MOESM2_ESM.pdf]

nature portfolio

Corresponding author(s): Stephen R. Quake

Last updated by author(s): Jan 23, 2022

Reporting Summary

Nature Portfolio wishes to improve the reproducibility of the work that we publish. This form provides structure for consistency and transparency in reporting. For further information on Nature Portfolio policies, see our Editorial Policies and the Editorial Policy Checklist.

Statistics

For all statistical analyses, confirm that the following items are present in the figure legend, table legend, main text, or Methods section.

n/a	Confirmed	
☐	☒	The exact sample size (*n*) for each experimental group/condition, given as a discrete number and unit of measurement
☐	☒	A statement on whether measurements were taken from distinct samples or whether the same sample was measured repeatedly
☐	☒	The statistical test(s) used AND whether they are one- or two-sided<br>*Only common tests should be described solely by name; describe more complex techniques in the Methods section.*
☐	☒	A description of all covariates tested
☐	☒	A description of any assumptions or corrections, such as tests of normality and adjustment for multiple comparisons
☐	☒	A full description of the statistical parameters including central tendency (e.g. means) or other basic estimates (e.g. regression coefficient) AND variation (e.g. standard deviation) or associated estimates of uncertainty (e.g. confidence intervals)
☐	☒	For null hypothesis testing, the test statistic (e.g. *F*, *t*, *r*) with confidence intervals, effect sizes, degrees of freedom and *P* value noted<br>*Give P values as exact values whenever suitable.*
☒	☐	For Bayesian analysis, information on the choice of priors and Markov chain Monte Carlo settings
☐	☒	For hierarchical and complex designs, identification of the appropriate level for tests and full reporting of outcomes
☒	☐	Estimates of effect sizes (e.g. Cohen's *d*, Pearson's *r*), indicating how they were calculated

Our web collection on statistics for biologists contains articles on many of the points above.

Software and code

Policy information about availability of computer code

Data collection	No software was used.
Data analysis	Bioinformatic processing: For each sample, raw sequencing reads were trimmed using Trimmomatic (v 0.36) and then mapped to the human reference genome (hg38) with STAR (v 2.7.3a). Duplicate reads were then removed by GATK's (v 4.1.1) MarkDuplicates tool. Finally, mapped reads were sorted and quantified using htseq-count (v 0.11.1) generating a counts table (genes x samples). Read statistics were estimated using FastQC (v 0.11.8). Across samples, the bioinformatic pipeline was managed using Snakemake (v 5.8.1). Read and tool performance statistics were aggregated across samples and steps using MultiQC (v 1.7).<br>Data analysis: Analyses were performed in Python (v 3.6) or R (v 3.5). In python, we used Scikit-learn (v 0.23.2), Scipy (v 1.5.1), nheatmap (v 0.1.4), Kneed (v 0.7.0), Seaborn (v 0.10.0), and GProfiler (v1.0.0). In R, we used Limma (v 3.38.3).<br>All code are available on Github at https://github.com/miramou/pe_cfrna.

For manuscripts utilizing custom algorithms or software that are central to the research but not yet described in published literature, software must be made available to editors and reviewers. We strongly encourage code deposition in a community repository (e.g. GitHub). See the Nature Portfolio guidelines for submitting code & software for further information.

Data

Policy information about availability of data

All manuscripts must include a data availability statement. This statement should provide the following information, where applicable:

- Accession codes, unique identifiers, or web links for publicly available datasets
- A description of any restrictions on data availability
- For clinical datasets or third party data, please ensure that the statement adheres to our policy

Raw and processed sequencing data (BioProject PRJNA792450) are available via the SRA (SRP352519) and GEO (GSE192902), respectively. Data were mapped using the human reference genome (hg38) and annotated using ENSEMBL version 82. We used publicly available data from the HPA (v19, https://v19.proteinatlas.org/), TSP (v1.0, https://tabula-sapiens-portal.ds.czbiohub.org/), Gene ontology: biological processes and cellular compartments (GO:BP, GO:CC, released 2021-05-01), Reactome (REAC, released 2021-05-07), and Kyoto Encyclopedia of Genes and Genomes (KEGG, released 2021-05-03), and publications 43-48.

Field-specific reporting

Please select the one below that is the best fit for your research. If you are not sure, read the appropriate sections before making your selection.

☒ Life sciences ☐ Behavioural & social sciences ☐ Ecological, evolutionary & environmental sciences

For a reference copy of the document with all sections, see nature.com/documents/nr-reporting-summary-flat.pdf

Life sciences study design

All studies must disclose on these points even when the disclosure is negative.

Sample size	To identify changes associated with PE well before traditional diagnosis, we designed a prospective study and recruited pregnant mothers at their first clinical visit to Stanford's Lucile Packard Children's Hospital, between 5–12 weeks of gestation, of which 131 were included in this study (94 NT, 37 with PE) prior to sample QC. For each participant, we analyzed cfRNA for samples collected at multiple time points. We also obtained samples from an independent cohort collected at several independent institutions (Validation 2), which consisted of 89 samples collected prior to 16 weeks of gestation from 87 mothers (61 NT, 26 PE).<br>For Discovery and Validation 1, we included all enrolled mothers who developed PE and had at least 1 mL banked blood plasma sample remaining. For Validation 2, we included all enrolled mothers who developed PE and had at least 1 mL banked blood plasma sample collected at ≤16 weeks of gestation remaining. In all cases, we chose a case:control ratio of approximately 1:2 to increase statistical power. We also ensured that case and control groups were matched for race and ethnicity. No other matching or exclusion criterion were used, and we performed no further sample selection prior to sample processing.
Data exclusions	Our final analysis included a subset of the available samples that passed pre-defined quality metrics as discussed in the Supplement (Supp. Note 1, Methods, Extended Data Fig. 1). Quality control was performed on a per sample as opposed to per subject basis and so subjects had a variable number of samples that passed QC (Supplementary Table 1).
Replication	To verify our results, we split our larger Stanford cohort into two groups (Discovery, Validation 1) and further replicated any reported findings using 2 independent cohorts (Validation 2, Del Vecchio). The larger Stanford cohort was split into Discovery (n = 88, [60 NT, 28 with PE]) and Validation 1 (n = 43, [34 NT, 9 with PE]) cohorts. We also further replicated our results using 2 independent cohorts: Validation 2 (n = 87, [61 NT, 26 with PE]) and Del Vecchio (n = 22, [8 NT, 5 with PE, 7 with gestational diabetes, 2 with chronic hypertension]), a previously published cohort processed by a completely separate team (Del Vecchio et al. 2020). In all cases, we successfully replicated our initial findings.
Randomization	To allocate samples to the control group (normotensive) or case group (preeclamptic), we relied on current clinical guidelines. Specifically, we defined a pregnancy as normotensive if it was both uncomplicated and went to full-term (≥ 37 weeks) and as preeclamptic based on current clinical guidelines (see Methods). We also explicitly tested for covariate differences between both groups and describe this both in the main text (see section titled Clinical study design, Fig. 1) and extended data (see Methods, Extended Data Table 1).<br>To split our larger Stanford cohort into Discovery and Validation 1, we first allocated samples using sequencing batch of which there were 3. We allocated the sequencing batch with the most PE samples to Discovery to ensure sufficient statistical power and the second most PE samples to Validation 1. Sequencing batches themselves contained randomly allocated samples based on subject ID such that all samples from the same subject were in the same batch. For the final sequencing batch, we randomly allocated subjects to either Discovery or Validation 1 such that all samples from 1 subject were part of the same group (either Discovery or Validation 1) and we maintained at least a 1:2 case to control ratio in both groups.
Blinding	Investigators were blinded during data collection as pregnancy outcomes were not known yet. Investigators were not blinded during data analysis as analysis methods required knowledge of outcome (i.e., supervised learning).

Reporting for specific materials, systems and methods

We require information from authors about some types of materials, experimental systems and methods used in many studies. Here, indicate whether each material, system or method listed is relevant to your study. If you are not sure if a list item applies to your research, read the appropriate section before selecting a response.

Materials & experimental systems

n/a	Involved in the study
☒	☐ Antibodies
☒	☐ Eukaryotic cell lines
☒	☐ Palaeontology and archaeology
☒	☐ Animals and other organisms
☐	☒ Human research participants
☒	☐ Clinical data
☒	☐ Dual use research of concern

Methods

n/a	Involved in the study
☒	☐ ChIP-seq
☒	☐ Flow cytometry
☒	☐ MRI-based neuroimaging

Human research participants

Policy information about studies involving human research participants

Population characteristics

The cohorts described herein are composed of pregnant mothers (aged 18 years or older) of varied ethnicity, race, age, and BMI among other pre-pregnancy and pregnancy characteristics (Results "Clinical study design", Fig. 1, Extended Data Table 1).

Recruitment

Discovery and Validation 1 were collected as part of a longitudinal, prospective study at Stanford University. We enrolled pregnant mothers (aged 18 years or older) receiving routine antenatal care on or prior to 12 weeks of gestation at Lucile Packard Children's Hospital at Stanford University, following study review and approval by the Institutional Review Board (IRB) at Stanford University (21956). All signed informed consent prior to enrollment. Mothers who agreed to participate in the study after enrollment at their first clinical visit were asked to complete a questionnaire available in multiple languages. Samples were then collected at routine care visits.
Validation 2 was collected as part of the Global Alliance to Prevent Preterm and Stillbirth (GAPPS) Pregnancy Biorepository at Yakima Valley Memorial Hospital, Swedish Medical Center, and the University of Washington Medical Center under review and approval by Advarra IRB (CR00195799). Mothers were enrolled during routine clinical care visit and then asked to fill out three questionnaires. Samples were drawn during routine care visits, and stored appropriately. Samples were processed and sequenced at Stanford under the same IRB as above (21956). All signed informed consent prior to enrollment.
In all cohorts, we recruited mothers who came in for routine care. Consequently, participant characteristics in the study reflect the socioeconomic, racial, and ethnic makeup of the larger area around the recruitment site. This presents a possible bias, which we focused on mitigating by reproducing our results using blood samples collected at different institutions (Validation 1, Validation 2, and Del Vecchio) with correspondingly distinct patient populations.

Ethics oversight

Institutional Review Board at Advarra (CR00195799) and Stanford University (21956)

Note that full information on the approval of the study protocol must also be provided in the manuscript.
